# Dynamic vibration-driven feedback shapes predator–prey interactions in an orb-weaving spider

**DOI:** 10.1371/journal.pcbi.1014802

**Published:** 2026-09-15

**Authors:** Hsin-Yi Hung, Abel Corver, Andrew Gordus

**Affiliations:** 1 Solomon H. Snyder Department of Neuroscience, Johns Hopkins University, Baltimore, Maryland, United States of America; 2 Department of Biology, Lund University, Lund, Sweden; 3 Department of Biology, Johns Hopkins University, Baltimore, Maryland, United States of America; University of California Santa Barbara, UNITED STATES OF AMERICA

## Abstract

Animals flexibly adjust their movements in real time to capture prey using environmental sensory cues. While sensorimotor transformations have been extensively studied in visual and somatosensory systems, their structure remains poorly understood in substrate-borne vibration sensing. Here, we combined high-resolution web vibration recordings, capable of resolving micrometer-scale displacements, with fine-scale behavioral tracking in the orb-weaving spider *Uloborus diversus* to investigate how vibration sensing guides prey capture. Using unsupervised modeling, we identified distinct behavioral states and their typical temporal sequence during capture. Predictive generalized linear models revealed reciprocal predator–prey dynamics: when *Drosophila* vibrations weakened, spiders often switched to actions such as crouching or shaking that enhanced signal detectability. These active phases were followed by static phases marked by increased pure fly-induced vibration power, consistent with an active sampling strategy. Conversely, flies tended to freeze during spider movement and struggled when the spider was still. Spiders also reliably oriented toward the web radius with the highest vibrational amplitude, indicating an amplitude-based strategy for prey localization. Together, our findings reveal a structured sensorimotor transformation linking external vibration cues to the spider’s behavioral choices, forming a dynamic feedback loop between vibratory stimulus and movement. This work uncovers general principles of active sensing and closed-loop control in a non-visual invertebrate and suggests that similar strategies may underlie sensorimotor control across diverse animal systems.

## Author summary

Orb-weaving spiders rely on vibrations traveling through their webs to detect and locate prey. These signals, however, can be weak or change as prey struggle. We studied whether the *Uloborus diversus* adjusts its own movements to improve how it senses these vibrations. Using high-speed video to measure web motions and machine-learning tools to track spider body posture, we examined how spiders respond when prey-generated signals fluctuate. We discovered that spiders do not passively wait for signals; instead, they engage in a dynamic feedback loop with their prey. When vibrations from the fly weaken, the spider triggers specific movements—crouching or shaking—which actively amplify the prey’s signal. These actions are followed by precise orientation toward the prey. Our findings suggest that spiders can influence the sensory information available to them, rather than relying solely on passive detection. This work provides insight into how animals with simple nervous systems may use movement to shape their perception of the environment.

## Introduction

Animals continuously gather information from their environment through sensory systems to guide context-dependent behaviors such as foraging [1,2] and courtship [3,4]. Sensory signals, however, are often noisy or transient, particularly during rapid or dynamic interactions such as prey capture. To overcome this, many species employ active sensing strategies [5,6]—coordinated motor actions that enhance the acquisition of behaviorally relevant stimuli. Active sensing has evolved across sensory modalities, including saccades in primate vision [7], echolocation in bats [8,9] and whales [10] (audition), sniffing in rodents [11] (olfaction), tail movements in electric fish [12,13] (electrosensation), and whisking or antennal scanning in insects and rodents [14–18] (mechanosensation).

Among mechanosensory systems, vibration-based sensing represents a particularly rich substrate for active strategies, as mechanical signals can be shaped both by the animal’s body posture and by the physical properties of the sensory medium itself. *Uloborus diversus*, an orb-weaving spider that naturally constructs horizontal webs, offers a powerful model for investigating vibration-based sensorimotor strategies during naturalistic prey capture. Unlike many animals that rely on vision or audition, orb-weaving spiders primarily detect web-borne vibrations through slit sensilla on their legs, enabling them to localize and evaluate struggling prey with high sensitivity [19,20]. Yet the extent to which spiders actively modulate this sensory medium remains less understood.

Recent findings show that some species, such as *Hyptiotes*, can actively generate and release tension to amplify mechanical power during prey capture [21]. Other orb-weaving spiders also perform stereotyped motor actions during prey capture [21,22]—including crouching and leg-lifting—that likely alter their mechanical coupling to the web. Together, these observations suggest that the web functions not only as a sensory substrate [21] but also as a dynamically tunable mechanical interface, whose tension and vibratory transmission properties can be modulated by the spider’s postures [19,23–25] and movements [26–28]. Despite this tight biomechanical coupling, it remains unclear whether the spider’s actions and the prey’s struggling are random or instead embedded within a structured sensorimotor loop. In particular, the vibratory cues that trigger specific spider actions—and how those actions subsequently reshape the vibratory input from the prey—are poorly understood.

Here, we focused on interactions involving web-caught prey, whose vibrations typically peak at 5–50 Hz [19,29], to investigate how spiders and prey influence each other through web-transmitted vibrations. We tested whether *U. diversus* uses specific motor behaviors as active sensing strategies to enhance the detection of prey-induced signals. First, we used high-resolution, video-based tracking to map the whole-web vibratory landscape during spider–prey interactions with sub-pixel sensitivity, resolving displacements as small as 7.1 µm. Classical behavioral measurements in other orb-weaving spiders (e.g., *Zygiella*, *Nephila*) show that prey-capture responses are triggered by silk displacements of 100–1000 µm at low frequencies (<100 Hz) [22]. Moreover, electrophysiological measurements indicate that slit sensilla exhibit sensitivities on the order of ~10 µm below 40 Hz [20], indicating the vibrations quantified here are well within the biologically effective range for orb weavers. Next, we applied unsupervised behavioral state discovery to leg kinematics to identify recurring motor patterns, establishing a quantitative framework for the spider’s behavioral repertoire. Analysis of state transitions revealed that vibratory signals from *Drosophila* increased following the spider’s crouching and shaking. Using generalized linear models (GLMs) to link vibratory input to behavior, we found that decreases in prey vibrations predicted subsequent crouching and shaking, revealing a context-dependent feedback dynamic. Conversely, flies froze when spider joint-movement power increased and struggled when it decreased. Finally, the largest pixel-intensity changes along radial threads predicted spider orientation, highlighting spatial computation and closed-loop control in prey localization.

Together, our findings reveal a dynamic, bidirectional sensorimotor loop in *U. diversus* and provide the first quantitative framework linking substrate vibrations to discrete, predictive behavioral states in a non-model organism.

## Results

### Web-caught *Drosophila melanogaster* and *Drosophila virilis* produce low-frequency vibration of 2–50 Hz on the web

To investigate the spider’s vibration-based sensorimotor transformation, we first defined the stimulus landscape generated by web-caught prey that drives prey capture behavior. Previous studies using traditional laser Doppler vibrometry showed that entrapped insects produce vibratory peaks between 5–30 Hz [29]. Although laser Doppler vibrometry provides [29,30] excellent amplitude resolution at micrometer scale, it measures vibration at only one or a few points, limiting its ability to capture spatially distributed vibration patterns across the entire web during spider–prey interactions. To overcome this limitation, we employed a high-resolution video-based recording system that enables analysis of whole-web vibrations through pixel-intensity changes [31,32].

**Fig 1:**
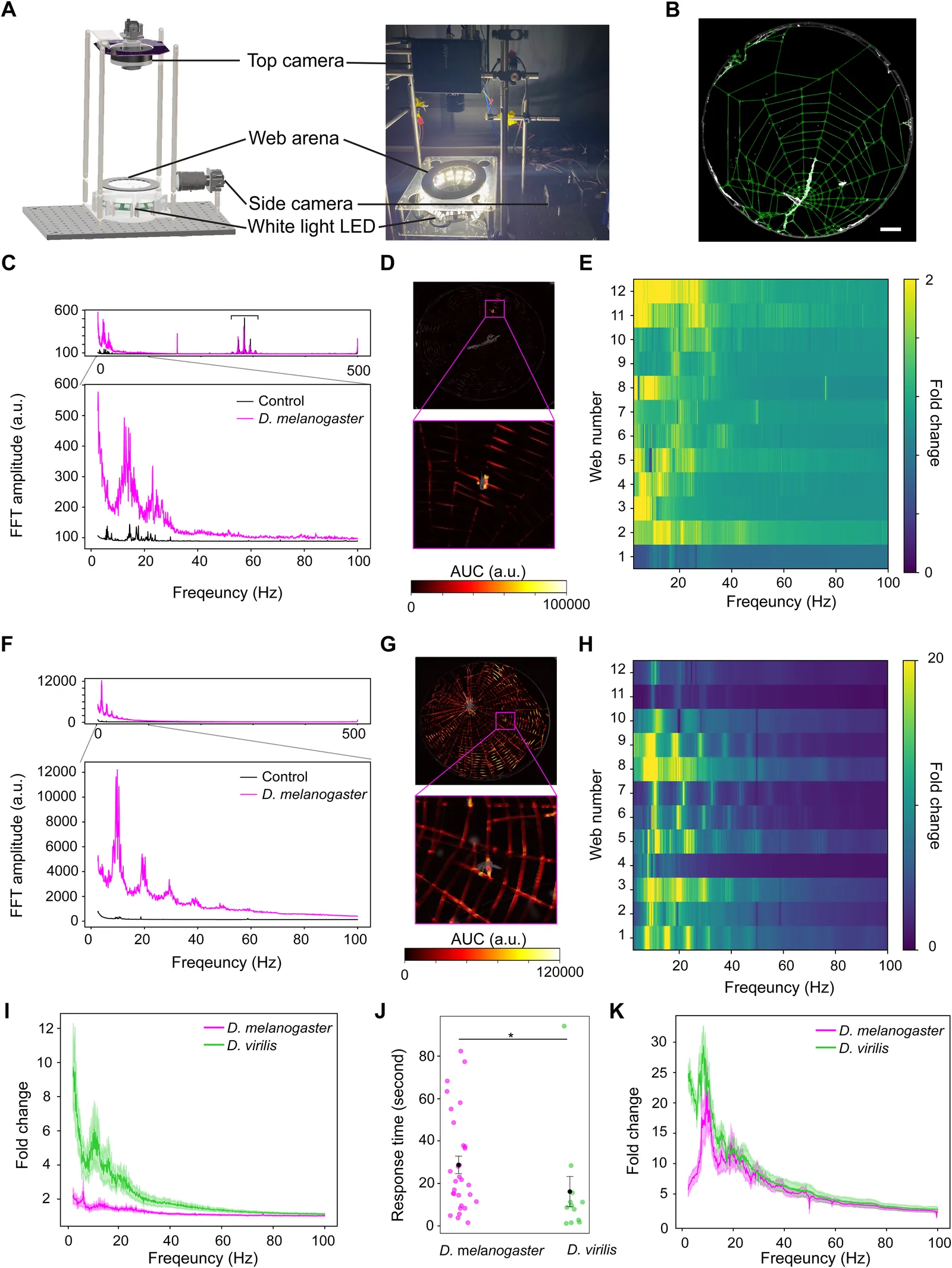
Web-borne vibrations induced by *Drosophila melanogaster* and *Uloborus diversus.* (A) Schematic of the experimental setup for simultaneous recording of spider behavior and web perturbations. A ring of white LEDs illuminates the silk, while a high-speed top-view camera (1000 Hz) captures web vibrations and a side-view camera (100 Hz) records spider behavior. (B) Example image showing annotated web architecture (green lines) used for vibration quantification. Scale bar = 1 cm. (C) Representative FFT traces of vibrations induced by *D. melanogaster* (magenta) and in the absence of the fly (control, black), revealing broadband low-frequency activity with power concentrated between 2–50 Hz. Bracket region denotes narrowband noise frequencies. (D) AUC map demonstrates that vibratory signals are spatially localized around the position of *D. melanogaster*. (E) Fold change, calculated as the ratio of FFT power in the experimental versus control conditions, across 12 independent webs, confirms consistent *D. melanogaster*-induced 2–50 Hz vibration. (F) *U. diversus* actively generates web vibrations during prey capture (magenta), producing resonance with a fundamental frequency at 10 Hz. (G) AUC maps during spider-induced vibrations reveal widespread vibratory activity across the entire web. (H) Fold change of spider-generated vibrations shows consistent harmonic peaks across 12 independent webs, highlighting the structured and resonant nature of self-generated signals. (I) Quantification of vibratory power reveals that *D. virilis* generates stronger signals than *D. melanogaster*, despite similar spectral range. The solid line indicates the mean fold change across 12 recordings; the shaded area represents the standard error of the mean (s.e.m.). (J) Total vibration power from both fly species in the presence of *U. diversus* (Solid line: mean fold change; shaded area: s.e.m.). (K) Response time of *U. diversus* when reaching *D. melanogaster* (28.79  ±  4.12 s, mean  ±   s.e.m.) versus *D. virilis* (16.20  ±  7.11 s, mean  ±   s.e.m.). A Mann–Whitney U test reveals a significant difference between groups (p = 0.0186).

We developed a custom behavioral recording system that simultaneously captured web vibrations using a top-view high-speed camera (1,000 Hz) and spider behavior using a side-view camera (100 Hz) (Fig 1A). The top-view recordings captured frequency components up to 500 Hz over 8.734-second sessions. A ring of white LEDs provided illumination to enhance silk contrast. Web geometry was annotated using a custom vision-based tracking algorithm (Fig 1B), and a U-Net model [33] trained on 91 manually labeled webs was used to predict web structure across additional recordings. Vibratory activity was quantified by measuring pixel intensity fluctuations [31,32] generated by light refracted through the silk, and Fast Fourier Transform (FFT) analysis [30,31,34] was applied to pixel intensity time series, enabling non-contact, high-resolution mapping of the full web’s vibratory landscape with fine spatial and temporal precision.

To validate this imaging approach, we delivered acoustic tones (100–500 Hz) to webs via speakers at three sound levels (61.8 ± 0.21, 67.6 ± 0.24, and 71.6 ± 0.35 dB, mean ± s.e.m.; n = 4 webs × 5 frequencies). The pixel-intensity method reliably captured amplitude differences and resolved multiple frequency components below 500 Hz (S1A–S1D Fig), with higher frequencies exhibiting reduced vibration amplitudes in the FFT spectra. As expected from aliasing theory, the 500 Hz stimulus — near the Nyquist limit of the camera sampling rate — produced no resolvable power in the FFT spectra (S1B-S1D Fig). Notably, subpixel vibrations as small as 7.1 µm, induced by a calibrated piezo stimulation (S1E Fig), were also detectable because thread motion modulates refracted light paths, producing intensity changes proportional to physical silk displacement rather than being constrained strictly by pixel size [32].

Having confirmed the sensitivity and frequency range of the recording system, we next characterized the vibratory signatures generated by web-caught prey. We first recorded a baseline control for each web in the absence of any stimulus, followed by an experimental condition in which a *D. melanogaster* (0.0016  ±  0.0002 g, mean  ±  **s.e.m.**; n = 24) was placed on the web. A representative example is shown in Fig 1C, where *D. melanogaster* generates broad-band, low-frequency vibrations between 2–50 Hz. To further verify that these vibrations originated from the fly’s movements, we calculated the area under the Fourier transform curve (AUC) along each silk thread. We observed that AUC values peaked around the fly’s location, indicating that these signals primarily arise from *D. melanogaster* activity rather than background noise (Fig 1D). We also computed the short-time Fourier transform (STFT) to examine the temporal patterns of prey signals, which exhibited irregular fluctuations (S1 Video). Next, we compared vibratory responses across 12 webs by computing the fold change (Equation 2) as the ratio of the Fourier power spectrum in the experimental condition to that in the control (Fig 1E). We found that *D. melanogaster* consistently produced broadband low-frequency signals in the 2–50 Hz range.

In a subset of recordings, we detected narrowband noise peaks in both control and experimental conditions (bracket in Fig 1C). Since these peaks were also present in the control and exhibited relatively low power (<600), we interpret them as likely artifacts, possibly arising from environmental or mechanical sources, rather than biologically relevant signals. Indeed, when we plotted the AUC map between 270–300 Hz, it revealed uniformly distributed power across the entire web (S1F Fig). The corresponding STFT further confirmed that this artifact persisted throughout the recording (S1G Fig), in contrast to the localized and irregular patterns characteristic of prey-induced vibrations.

To assess species-specific differences in vibratory signaling, we repeated the experiment using *Drosophila virilis* (0.0022  ±  0.0002 g, mean  ±   **s.e.m.**; n = 24), a larger species of *Drosophila* [35] (S2A Fig). Despite exhibiting a similar spectral range (S2B-S2D Fig), *D. virilis* generated substantially greater vibratory power on the web (Fig 1I). These findings indicate that differences in web-borne signals between the two *Drosophila* species arise primarily from vibratory intensity rather than frequency composition.

### Spiders actively produce movements, inducing harmonic vibrations on webs, in response to *D. melanogaster*-generated cues

To determine whether spiders actively modulate web-borne vibrations in response to prey, we placed both *D. melanogaster* and *U. diversus* on the web. As a baseline, we first recorded vibratory activity with a stationary *U. diversus* in the absence of prey. Under these conditions, no significant vibrations were detected, indicating minimal background activity from the spider or ambient air currents (Fig 1F, black). Upon the introduction of *D. melanogaster*, however, spiders actively generated vibrations (Fig 1F, magenta), suggesting an active sensing mechanism triggered by the presence of prey. The AUC map revealed widespread vibration power across the entire web during active sensing (Fig 1G). Notably, these spider-induced movements produced harmonic vibrations with a fundamental frequency of approximately 10 Hz. This pattern was consistently observed in 11 of 12 recordings (Fig 1H), regardless of spider–prey distance (3.28 ± 0.51 cm, mean ± s.e.m., n = 12; S2H Fig, magenta), with the spider typically positioned at the web’s center (web diameter = 10 cm).

Whether this 10 Hz frequency reflects the web’s natural resonance was examined using a control experiment in which a 3D-printed object (0.0159 g) matched to the spider’s body mass (0.0160 ± 0.0015 g, mean ± **s.e.m.**; n = 48) was place on the web and silk vibrations were measured after mechanically perturbing the object. The web exhibited a pronounced 10 Hz amplitude (S1H-S1I Fig), confirming that this frequency corresponds to the web’s natural resonance. Larger movements of the 3D-printed object also induced harmonics. These results suggest that during prey capture, spiders actively generate vibrations and may actively or passively move with the web at its resonant frequency, potentially amplifying vibratory signals.

To probe whether this response depends on prey signal strength, we repeated the experiment with *D. virilis*, which generates higher-amplitude vibrations (Fig 1I). As predicted, spiders responded more rapidly to *D. virilis* than to *D. melanogaster* (Fig 1J). Notably, unlike the consistent harmonics observed with *D. melanogaster*, harmonic peaks at 25, 50, and 75 Hz were detected in only one of 12 trials with *D. virilis* (Figs 1K, S2E-S2G). This difference is unlikely to reflect variation in spider–prey distance, as no significant difference in distance was observed between the two groups (S2H Fig).

Together, these findings suggest that *U. diversus* actively produces large movements in response to weak vibratory cues, inducing harmonics that may enhance signal detectability via resonance. In contrast, when interacting with prey that already produces strong vibratory signals (*D. virilis*), the spider does not generate harmonics and reaches the prey faster.

### Unsupervised modeling reveals structured state transitions and timing modulation in spider prey capture across species

**Fig 2:**
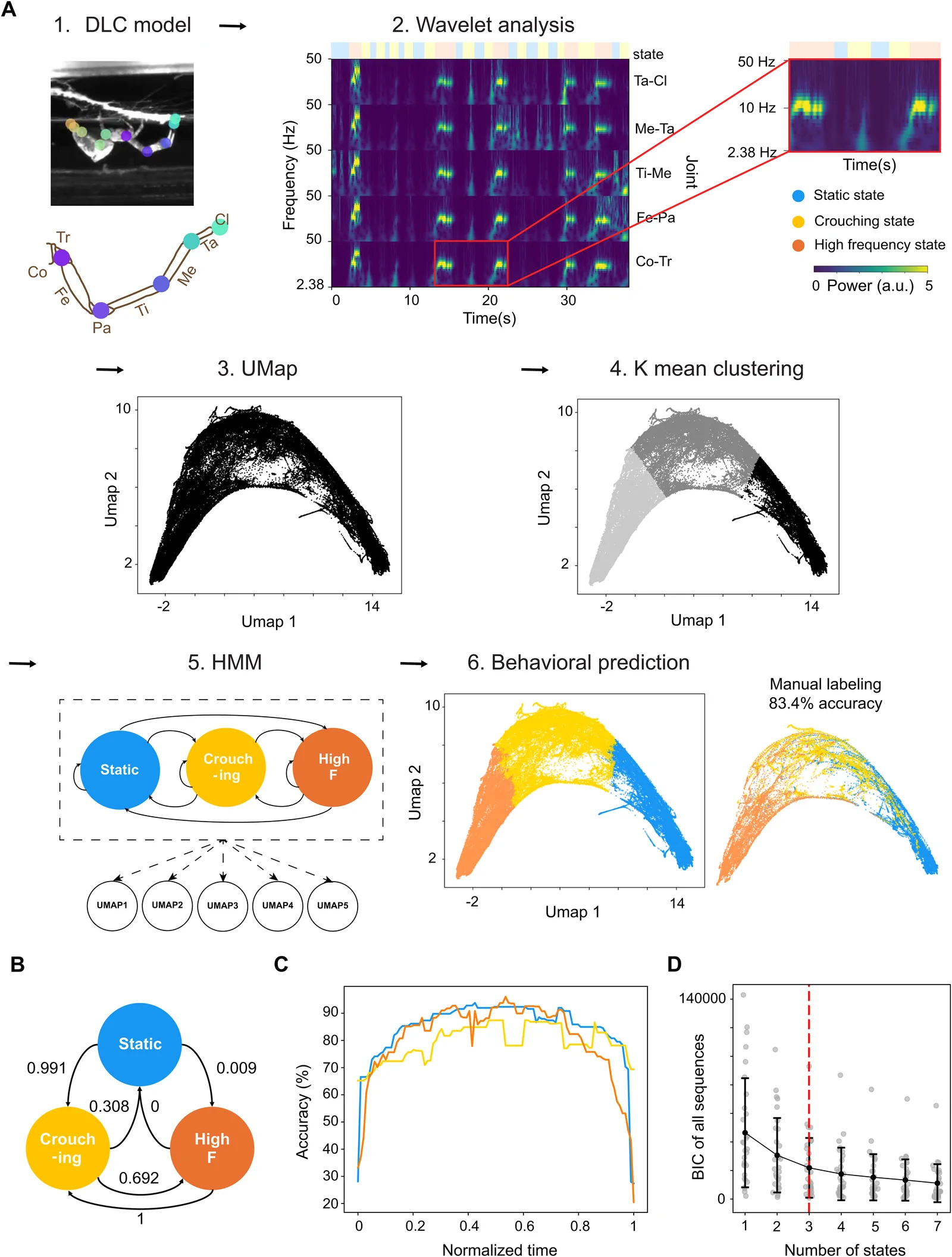
Unsupervised identification of spider prey capture dynamics during interactions with *D. melanogaster* using behavioral state modeling. (A) Overview of the behavioral analysis pipeline. (1) Side-view recordings were analyzed with DeepLabCut to track 20 leg joints. Diagram denotes the joints tracked. (2) Wavelet spectrograms were computed from five joints on the left anterior leg (left), with a magnified view of the red-highlighted segment (right). Ethogram above the spectrogram denotes behavioral states. (3) UMAP was applied to reduce the joint dynamics to five dimensions across 30 recordings (UMAP dimensions 1 & 2 are shown). (4) Unsupervised K-means clustering in the UMAP space identified three distinct behavioral clusters. (5) A HMM was then trained using the cluster-derived probability distribution to model the emission probabilities. (6) The HMM predicted behavioral states across all 30 videos, achieving 83.4% accuracy on 10 manually annotated sessions. Anatomical diagram: Co: Coxa, Tr: Trochanter, Fe: Femur, Pa: Patella, Ti: Tibia, Me: Metatarsus, Ta: Tarsus. (B) HMM-inferred state transition probability matrix after removing self-transition. (C) Time-normalized prediction accuracy of each state. Decreased accuracy is observed near state transitions, reflecting increased temporal uncertainty during behavioral switching. (D) BIC for HMMs with increasing numbers of hidden states. A sharp decrease from one to three states is observed, after which improvements plateau, supporting the selection of a three-state model. Dark grey circles represent individual sequences (30 videos). Data are presented as mean  ±  s.d. (filled circles and bars).

Having characterized the vibratory landscape of prey capture, we next examined how spiders coordinate their movements during prey capture. Spider leg movements were tracked from side-view camera recordings (100Hz; Fig 1A) using DeepLabCut [36,37], which identified five joints on each of the two anterior and two posterior legs (20 joints total). To extract stereotyped movement [38–40] during highly dynamic and non-stationary prey capture behavior, wavelet analysis was then applied to quantify the spiders’ limb movements (Fig 2A). From the joint wavelet spectrum, three behavioral states were observed, which we named static state, crouching state, and high-frequency state. The static state was defined by low power in the spectrum due to very little limb movement (S2 Video). When the spider crouched, the joints moved in the 3–10 Hz frequency range. During high-frequency shaking states, the joint wavelet spectrum exhibited a prominent peak at 10 Hz—closely matching the resonance frequency identified in top-view recordings. This state could occur while the spider remained in one location on the web, or during walking or turning.

To automatically classify these states, we developed an unsupervised modeling pipeline combining nonlinear dimensionality reduction, clustering, and Hidden Markov modeling (HMM) to predict a spider’s behavioral states based on joint wavelets. We focused on five joints of the left anterior leg, as the four legs exhibit similar wavelet patterns during prey capture (S3A Fig) and the anterior legs serve as the primary motor output. We then applied Uniform Manifold Approximation and Projection (UMAP) [41] dimensionality reduction across 30 spiders, yielding a five-dimensional nonlinear embedding. Then, we applied unsupervised K-means clustering to the UMAP space, identifying three clusters in behavioral continuum space to extract the probability distribution of three states. A HMM was constructed using the five-dimensional UMAP embeddings as observables, with the emission probability distribution determined by K-means clustering [42].

This unsupervised HMM enabled moment-to-moment behavior prediction, achieving 82.9% accuracy across 10 manually annotated trials out of 30 recordings with *D. melanogaster* interactions. Our HMM revealed a high probability of self-transitions within each behavioral state during prey-capture dynamics (S3B Fig). To better visualize the transitions between distinct states, we excluded self-transitions and normalized the remaining state-to-state transition probabilities (Fig 2B). Notably, the model predicted all high-frequency states are followed by crouching states, likely reflecting biomechanical constraints that prevent abrupt cessation of movement. Overall, model performance declines during state transitions, but achieves 70–100% accuracy during non-transition periods (Fig 2C).

To validate the number of behavioral states, we evaluated model performance using the Bayesian Information Criterion (BIC). BIC values dropped sharply between one and three states, followed by a plateau, indicating minimal improvement with additional states (Fig 2D). We further quantified BIC changes relative to both the preceding state model and the best-fitting model per sequence (S3C-S3D Fig). In both cases, the greatest improvement occurred at three states, with marginal gains thereafter. Together, these findings support a three-state model underlying spider prey capture dynamics.

To test the generalizability of this three-state classification pipeline, we applied it to recordings of spiders interacting with a second prey species, *D. virilis*, which produces comparable vibratory frequencies (Fig 1I). Joint wavelet data from 12 *D. virilis* trials were projected onto the *D. melanogaster*-derived UMAP embedding (S3E Fig), and a new HMM was trained within this shared low-dimensional space. Using four manually labeled sequences for validation, the *D. virilis* model achieved 82.9% accuracy, comparable to that of the *D. melanogaster*-trained model. Importantly, transition dynamics were also qualitatively similar across prey types (S3F Fig), suggesting conserved underlying sensorimotor structure and supporting the robustness of the modeling framework.

Given that *U. diversus* responds more rapidly to *D. virilis* (Fig 1J), we hypothesized that this behavioral efficiency might arise from reduced engagement in crouching or shaking, or from shorter durations within each behavioral state. While the number of static, crouching, and shaking events did not differ significantly between prey types (S3G Fig), state durations were significantly shorter during *D. virilis* interactions (S3H Fig). In particular, crouching dwell times were reduced (S3I Fig), suggesting faster behavioral transitions and a larger decay constant. Together, these results demonstrate that *U. diversus* exhibits structured and stereotyped motor dynamics during prey capture that are robust across prey types, yet flexibly modulated in timing by sensory input intensity.

### Spiders’ crouching and shaking increase vibratory stimulus power from *Drosophila melanogaster*

To uncover the functional role of crouching and high-frequency shaking during prey capture, we examined how these motor states influence vibratory signals from prey. As previously described, *D. melanogaster* produces relatively low-amplitude vibrations on the web (Fig 1I), and spiders exhibit significantly longer state durations during interactions with this prey compared to *D. virilis* (S3H-S3I Fig). We hypothesized that crouching and high-frequency shaking behaviors may actively modulate prey signals to amplify vibratory power on web.

**Fig 3:**
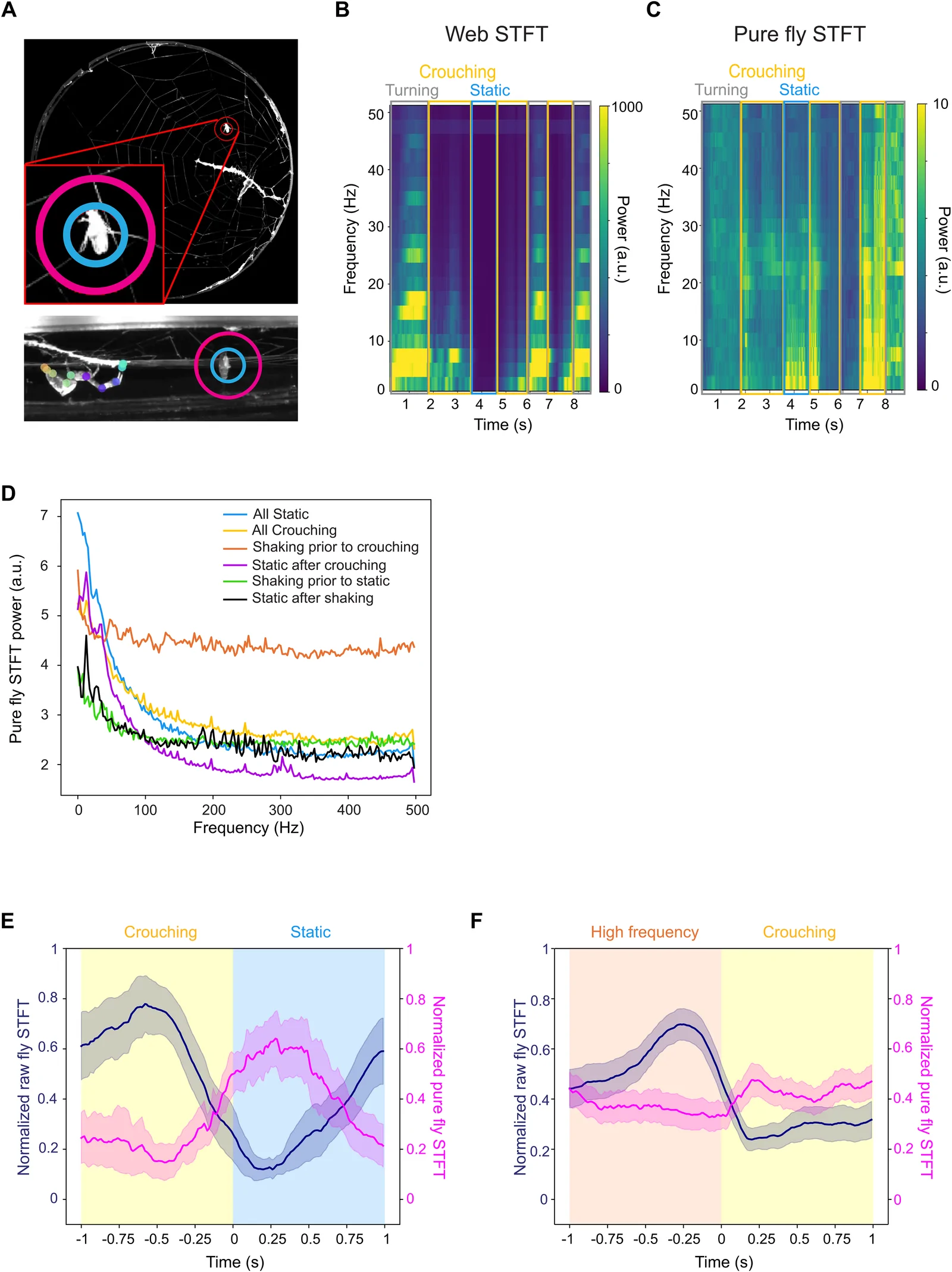
Spider crouching and high frequency movements increase sensory stimuli power from *Drosophila melanogaster.* (A) Schematic illustrating ROIs used to extract vibratory signals from the 1000 Hz top-view camera and behavioral states from the 100 Hz side-view camera. Area within the cyan circle was used to quantify the raw fly signal, which was normalized to signal between the magenta and cyan circles. (B) STFT power spectrum of the raw fly signal from one representative recording shows large amplitude fluctuations during spider movement, reflecting spider-induced web motion. (C) STFT spectrum of the pure fly signal from the same representative recording as in b shows minimal power during spider turning, indicating passive fly movement. By contrast, crouching and static states are associated with elevated spectral power, suggesting active fly movement. (D) Average STFT power of pure fly signal across 12 top-view recordings reveals distinct spectral profiles across behavioral states. (E) Quantification across 16 side-view recordings confirms consistent enhancement of low-frequency (0–30 Hz) normalized pure fly signal power during static states following crouching (n = 22). Shaded areas represent 95% confidence intervals. (F) Similar enhancement is observed during transitions from shaking to crouching crouching (n = 76), as predicted by the HMM. Shaded areas represent 95% confidence intervals.

Because spider movement itself induces substantial web vibrations (Fig 1F–1H), we sought to isolate prey-induced signals from those driven by the spider. To achieve this, we extracted STFT of pixel intensity fluctuations from silk threads in a region of interest (ROI) centered on the *D. melanogaster* and normalized these values by STFT of fluctuations in a surrounding peripheral area (Fig 3A). This normalization yielded a “pure” fly signal (Equation 7) from top-view recordings, isolating prey-specific motion independent of spider-induced noise. We manually annotated spider’s behavioral states in the top-view data because state predictions from the HMM were based on lower frame rate side-view recordings at 100 Hz.

Fig 3B and 3C shows a representative recording illustrating that the raw fly signal reflects the original fly vibrations, while the pure fly signal isolates prey-induced vibrations independent of spider movement. During large spider movements in high-frequency state, such as turning, the raw fly signal displayed large power (S3 Video), whereas pure fly signal exhibited low power (Fig 3C), indicating that the fly moved passively with the vibrating web. By contrast, crouching behavior induced greater pure fly signal power compared to high-frequency state. Strikingly, upon cessation of crouching, fly power further increased—exceeding levels observed during crouching—suggesting that the crouching behavior itself may trigger fly movement and enhance the resulting vibratory signal from *D. melanogaster*.

To quantify these effects across multiple *D. melanogaster* trials, we averaged STFT power over time from 12 top-view video recordings. In the pre-capture static state, fly-induced vibrations were predominantly low-frequency (<30 Hz; Fig 3D, cyan; n = 10). When the spider was crouching, the pure fly signal showed a spectral peak at 12 Hz (Fig 3D, straw; n = 40). Notably, power in the subsequent static state increased further, peaking at 12 Hz and 33 Hz (Fig 3D, purple; n = 17). Shaking behavior typically elicited broadband fly signals spanning 0–500 Hz, with shaking preceding crouching (Fig 3D, orange; n = 12) exhibiting higher power than shaking followed by a return to static (Fig 3D, light green; n = 6). Static states following crouching (Fig 3D, purple; n = 17) or shaking (Fig 3D, black; n = 6) consistently exhibited increased vibratory power relative to the prior state, suggesting that these behaviors enhance transmission of fly-induced signals across the web.

To examine whether enhanced pure fly signals in static states consistently increased following crouching throughout the entire prey capture sequence, we analyzed behavioral state transitions predicted by the HMM using extended-duration side-view recordings. We applied the same ROI-based analysis to quantify fly signals (S4A Fig); however, due to limited visibility of silk threads in the side view, we defined the normalized fly signal directly from pixel intensity of the fly itself, rather than silk fluctuations.

S4A Fig shows a representative recording. Averaging the STFT power within the 0–30 Hz frequency band revealed a pronounced increase in the pure fly signal during the static periods immediately following spider crouching (S4 Video). To systematically quantify pure fly–signal amplification, we identified all state-transition events across 16 recordings and normalized each state’s duration. As shown in Fig 3E, the mean pure fly signal increased after crouching (n = 22), indicating that the spider’s behavior likely induced additional prey movement. Although the HMM did not predict transitions directly from shaking to static states, high-frequency shaking movements were consistently followed by elevated vibratory signals during the subsequent crouching state (Fig 3F; n = 76), supporting the role of both crouching and shaking in amplifying prey-derived vibratory input.

Although the raw fly signal in S4A Fig shows large rotational body movements during the first ~3 seconds, such rotations alter pixel intensity broadly across the entire fly, causing the central and peripheral regions to change in a similar manner. Because the pure fly signal is computed as the ratio of these regions, the resulting relative change is modest, and large whole-body rotations therefore appear smaller in the pure fly signal. This represents a limitation of the current metric, which is more sensitive to localized translational pixel-intensity changes than to broad rotational movements (S4 Video). Importantly, despite this limitation, the first static epoch produced significantly higher pure-fly-signal power than all crouching and high-frequency shaking epochs (S4C-S4D Fig), indicating that the metric reliably captures fly-generated vibration events that are behaviorally relevant relative to spider actions.

### Predator–prey feedback in vibratory signaling

**Fig 4:**
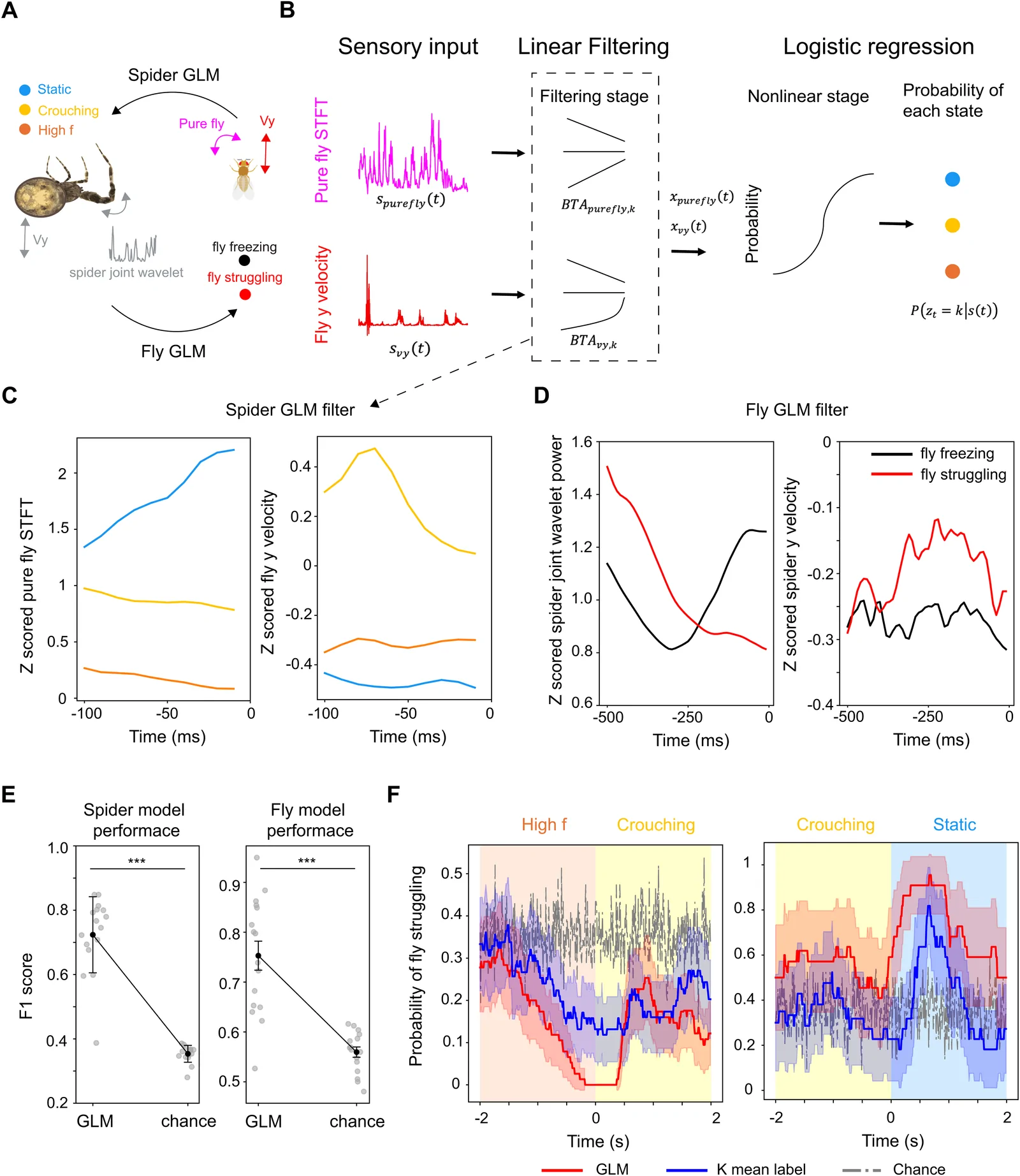
Sensorimotor models reveal predator–prey feedback. (A) Schematic of spider and fly GLMs. The spider GLM receives two sensory inputs from the fly: pure fly STFT power ratio (magenta) and vertical velocity (red). The fly GLM receives spider joint wavelet power and vertical velocity (grey). (B) Spider GLM predicts behavioral states from fly-derived sensory. Two features processed through BTAs to estimate linear filters. Filtered sensory inputs are passed through a logistic nonlinearity to compute the moment-to-moment probability of each state. (C) BTAs for the spider GLM showing sensory input aligned to spider behavioral state onsets (cyan: static; straw: crouching; orange: high-frequency movement). Spider BTAs show that static states follow increased fly vibration amplitude, whereas crouching and high-frequency movements follow decreased vibratory power (left). Crouching also follows reduced fly velocity (right). (D) BTAs for the fly GLM. Increased spider joint wavelet power precedes fly freezing (black, left), whereas decreased spider joint wavelet power precedes fly struggling (red). Larger deviations of spider velocity from its mean (black, right) are also associated with fly freezing. (E) GLMs outperform chance for both species (Mann–Whitney tests, ***p  <  0.001). (F) Fly struggling probability rises after termination of spider high-frequency movements (left) or crouching (right), consistent across k-means labels (blue) and GLM predictions (red). Shaded area represents 95% confidence interval.

If spider movements serve to amplify vibratory power from prey, we next asked whether prey-generated cues could, in turn, influence the spider’s behavioral transitions—and conversely, how spider movements affect prey behavior (Fig 4A). We analyzed 16 side-view recordings of spider–prey interactions and trained a multinomial GLM to classify spider behavior into three discrete states—static, crouching, and high-frequency movement—based on two features of *D. melanogaster*: the pure fly STFT power and fly vertical velocity (Fig 4B).

To examine how sensory input drives behavioral state transitions, we computed behavior-triggered averages (BTAs) — analogous to the spike-triggered average in neuroscience — by averaging sensory inputs over a 100 ms window preceding each state onset (Equation 14). This approach identifies the linear sensory signatures most predictive of each behavioral transition. The resulting BTA filters revealed that fly vibratory power increased prior to transitions into the static state, but decreased before crouching and high-frequency movements (Fig 4C, left). Crouching was also preceded by reductions in fly vertical velocity (Fig 4C, right). These patterns indicate that specific changes in prey-induced signals modulate the probability of entering distinct motor states: spiders tended to pause when flies were moving, and crouch when flies reduced their movements on the web.

We then projected raw sensory input onto the BTA filters via inner product (Equation 15–16), yielding filtered representations that capture the stimulus features most predictive of each state. These BTA-projected signals were then used as inputs to a multinomial logistic regression model (Equation 17) to estimate the probability of each behavioral state k given the sensory input (Fig 4B). Using 25% held-out data and 250 bootstrap iterations (n = 1000), the two-feature GLM significantly outperformed models using pure STFT power alone (p  <  10^-307^) or substituting horizontal for vertical velocity (p  <  10^-307^), and performed comparably to the three-feature model (p  =  0.683; S5A Fig). A 100-ms BTA window yielded optimal classification accuracy, with shorter (50 ms) and longer (250 ms, 500 ms) durations showing no improvement (S5B Fig). Also, filters derived from alternative window lengths produced similar trends (S5C Fig), indicating robustness of the sensory–behavior mapping.

Given that fly vibratory signals predict spider behavioral states, we next examined the reciprocal relationship — whether spider movements also influence prey behavior. We built a second GLM using mean joint wavelet power across five spider joints and spider vertical velocity as sensory inputs. Fly behavior was classified into “freezing” or “struggling” via K-means clustering of the pure fly STFT signal. Flies tended to struggle when spider joint wavelet power decreased, and freeze when it increased (Fig 4D, left). Larger deviations of spider vertical velocity from the mean were also linked to increased fly freezing (Fig 4D, right). Filters derived from alternative window lengths produced similar trends (S5D Fig), paralleling the robustness observed for the spider filters (S5C Fig). Together, these results support a dynamic feedback loop between predator and prey.

Both the spider GLM and fly GLM outperformed a chance model based solely on behavioral state distributions (Mann–Whitney tests, ***p < 0.001; Fig 4E). Finally, we found that fly struggling probability increased after spiders terminated high-frequency shaking, particularly during subsequent crouching phases (Fig 4F, left), whereas the chance model showed no change across state transitions. A similar increase occurred during crouching-to-static transitions (Fig 4F, right). These results were consistent with the enhanced prey vibratory power effects reported in Fig 3E and 3F.

### Spatial structure of vibratory input predicts orienting behavior

Previous studies [22,29,43–45] have suggested that spiders may use differences in vibration amplitude or timing across their legs to orient toward a signal source. Building upon this work, we examined whether video-based pixel-intensity changes, which are influenced by vibration amplitude, are sufficient to predict the spider’s orientation toward prey. If spider crouching and shaking enhance vibratory signals from *Drosophila*, this amplification should be detectable during subsequent static states.

**Fig 5:**
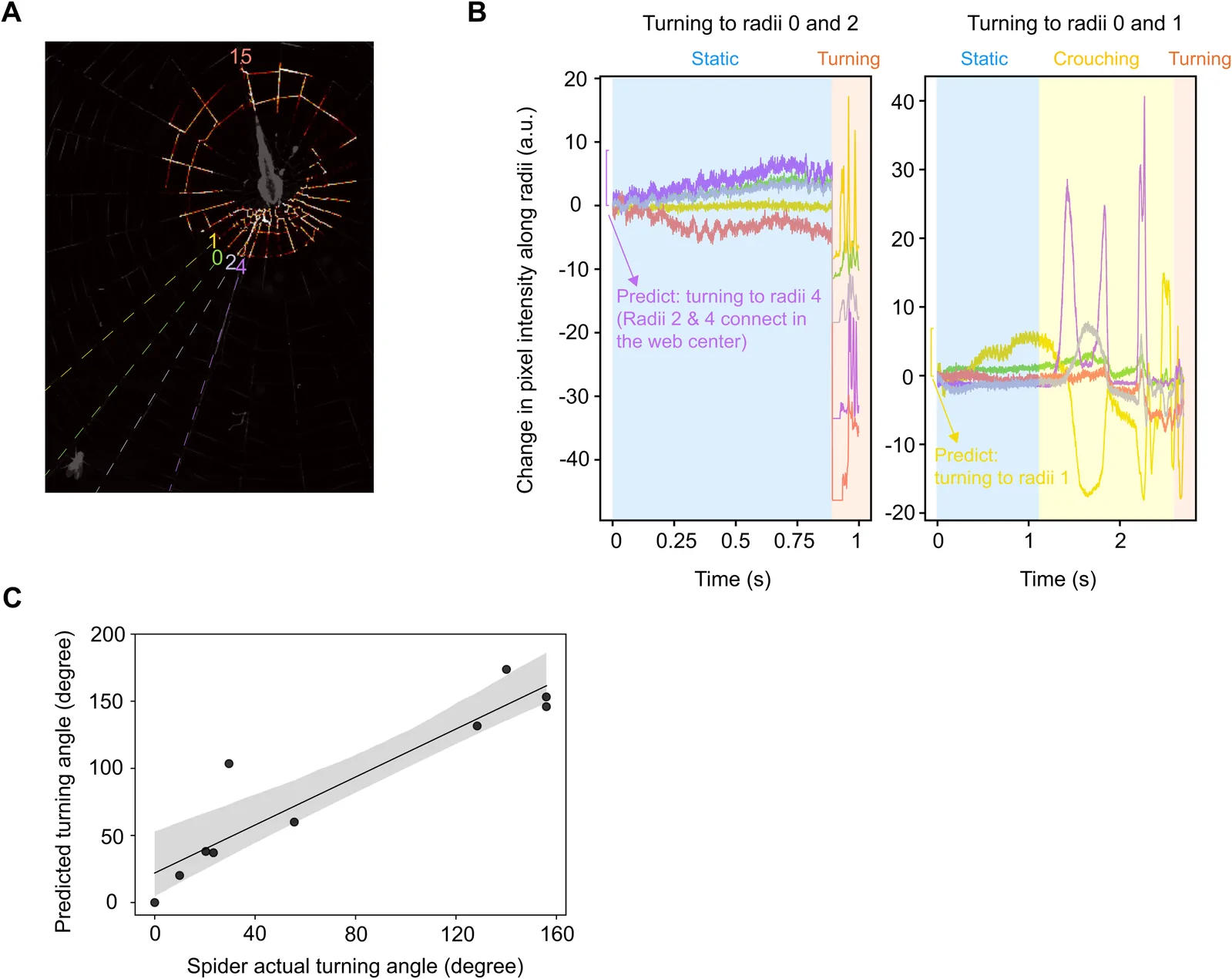
Spatially localized vibratory cues guide spider orienting behavior. (A) Schematic of analysis pipeline using top-view recordings to extract vibratory signals from individual radial threads prior to spider turning events. (B) Pixel intensity changes were quantified to estimate vibratory input along each thread within the spider’s peripheral sensory field. Two representative turning events are shown. Pixel intensity change is expressed relative to the static-state baseline, rather than as an absolute value, to account for variation in the angle of light reflection from the spider’s silk. Bracket indicates the largest-amplitude change during the static state preceding a turning event. Ethogram denotes spider behavioral states. (C) Across trials, the spider consistently turned toward the radial thread with the highest vibratory amplitude (p  = 1.027  ×  10^-4^, r  =  0.9289, slope  =  0.8943). A shaded area around the regression line represents the 95% confidence interval.

Using top-view recordings, we quantified vibratory signals by measuring pixel intensity changes along individual radial threads preceding each turning event (Fig 5A and S5 Video), assessing whether localized variations in signal amplitude across the spider’s peripheral sensory field could predict turning direction on a trial-by-trial basis.

For each turning event, we identified the radial thread exhibiting the greatest pixel intensity change — reflecting vibratory amplitude change— during the static state preceding turn onset, and compared its spatial position to the spider’s turning angle (Fig 5B). This analysis revealed a consistent correspondence: the location of peak vibration reliably predicted the direction of turning (Fig 5C). In nearly all cases, the spider turned toward the radial thread carrying the strongest relative vibratory signal.

These results indicate that spatially localized vibratory cues serve as directional guides during prey capture, suggesting that spiders extract positional information from the vibratory landscape of the web. This spatial encoding of sensory input provides additional evidence for a dynamic sensorimotor loop, in which environmental signals not only elicit behavior but are actively interpreted through structured motor responses. Our findings underscore the importance of peripheral sensory dynamics in guiding closed-loop prey localization.

## Discussion

Our study demonstrates that orb-weaving spiders engage in predictive sensorimotor strategies during prey capture, actively modulating web vibrations and adjusting their movements in response. Conversely, the prey also alter their behaviors based on the spider’s actions. This bidirectional feedback between spider and prey forms a dynamic loop in which motor actions reshape the vibratory environment and, in turn, guide subsequent behavioral transitions. By integrating high-resolution web recordings, unsupervised behavioral modeling, and interpretable stimulus–response analyses, we provide a quantitative framework for decoding active sensing in a non-model organism.

A key component of this sensorimotor loop is the spider’s ability to define and interpret web-borne prey signals. Although prey-generated vibrations can span a broad range (5–1000 Hz) [19], the signals produced by insects trapped in orb webs are typically concentrated at lower frequencies (5–50 Hz) [19,29] —a regime less explored than airborne [22,30] or rigid-substrate vibrations [46]. Using high-speed (1,000 Hz) vision-based tracking [31,32], we captured whole-web dynamics at the micrometer level resolution needed to map vibration patterns during prey capture. Vibrations produced by two *Drosophila* species fell within the expected 2–50 Hz band [19,29]. The similar frequency components shared by the two species may stem from comparable grooming frequencies: Ravbar et al. [47] reported that *D. melanogaster* grooming occurs at two timescales (5–7 Hz and 0.3–0.6 Hz), and Mueller et al. [48] demonstrated that five *Drosophila* species share core behavioral features, though they differ in the proportions of individual actions. While *D. virilis* was not included in that study, it likely shares similar grooming characteristics with *D. melanogaster*.

Beyond characterizing prey signals, we also examined how spiders respond when those signals are weak: spiders generated movements that induced oscillations near 10 Hz. This behavior aligns with prior work showing that similarly sized orb weavers oscillate at 10 Hz in both longitudinal and lateral directions [30], suggesting that spiders actively tune the web to enhance signal detectability. To understand how these vibratory cues map onto spider motor outputs, we next characterized the structure of spider prey capture behavior itself — identifying three states: static, crouching, and high-frequency movement. Like zebrafish prey capture [49], spider prey capture occupies a continuous motor space. The resulting classifications achieved 82.9% agreement with human annotations, without relying on subjective labeling. This approach extends fine-timescale state discovery—previously applied to flies [38] and web-making spiders [33]—to a naturalistic, vibration-evoked context.

Integrating these states with BTA adapted from the linear–nonlinear–Poisson (LNP) cascade model [50–53] and a GLM allowed us to predict moment-to-moment spider actions from vibratory input alone. This approach preserves predictive power while offering interpretability, computational efficiency, and robustness to local minima—a common issue in high-dimensional optimization. Notably, spiders initiated crouching and shaking when prey vibrations weakened, supporting an active-sampling strategy in which motor actions are deployed to probe or amplify weak signals [28]. These movements preceded static phases characterized by increased *Drosophila* vibration power, revealing a temporally structured sensorimotor loop akin to the feedback dynamics observed in *Drosophila* courtship [54]. This emphasizes closed-loop sensory guidance over passive mechanical properties [50–53] of the web to closed-loop sensory guidance during goal-directed behavior. High-resolution imaging further showed that spider orientation is reliably predicted by the radial thread with the highest vibratory amplitude, supporting a model of spatially selective, vibration-guided behavior.

Several limitations warrant consideration. First, our video-based method provides micrometer-scale spatial resolution, whereas spiders can detect nanometer-scale vibrations [19,20]. Importantly, classical prey-capture studies in other orb-weaving spiders (e.g., *Zygiella*, *Nephila*) report behavioral thresholds of 100–1000 µm for low-frequency (<100 Hz) web vibrations [21], suggesting that the micrometer- to millimeter-scale displacements observed in our recordings —including those we resolve down to ~7.1 µm—fall within the biologically responsive range for orb weavers, even though we cannot access the nanometer domain. Second, we did not examine airborne cues [30], which may contribute to prey detection in other species [55,56]. Ogre-faced spiders can detect airborne sound via web-mediated acoustic transduction [56], yet classical studies show that airborne cues are neither sufficient nor necessary to elicit prey-capture behaviors in *Zygiella* and *Nephila* [21], which occur robustly in response to silk vibrations alone. These findings reflect distinct sensory contexts: airborne cues support long-range detection, whereas prey capture following web contact depends on mechanical vibrations generated directly by the struggling prey. Consistent with this, we never observed prey-capture transitions prior to web contact in our experiments. Third, behavioral states contained subtypes—e.g., vigorous versus mild shaking—that may serve different functions. A parallel robophysical model [57] of spider crouching behavior demonstrated that vigorous shaking followed by crouching elicits distance-dependent prey echoes in a well-defined web structure. These findings suggest that the intensity and sequencing of behavioral subtypes may be functionally meaningful, and that propagation distance and web geometry modulate the vibratory signals received by the spider in ways not fully captured by our current measurements — motivating future work to characterize these subtypes in greater detail. Finally, crouching may also modulate web tension [26], introducing additional mechanical feedback not directly measured here.

A compelling question for future research is how postural configurations during static phases shape the mechanical tuning [23] and neural encoding of vibrations. In insects, joint angle–selective claw neurons and tonotopically organized club neurons have been shown to map limb position and vibration frequency [58,59], respectively. Whether spiders possess analogous encoding strategies—such as topographic maps of joint angle or frequency-selective afferents—remains an open question. Additionally, while central pattern generators (CPGs) orchestrate behaviors like walking and grooming in insects [60] and are modulated by sensory feedback [61], it is unclear whether spider prey capture involves similar architectures. Elucidating these mechanisms could uncover shared principles of sensorimotor coordination across species.

Overall, our findings offer valuable biological insights into the spider’s active vibration sensing during prey capture, and provide computational models for behavioral discovery and sensorimotor transformations. By combining dense kinematic measurements, unsupervised behavior discovery, and interpretable stimulus-response models, this work contributes to a growing understanding of how organisms integrate environmental input with motor plans. These results draw parallels to active sensing strategies observed in animals such as bats [62,63], rodents [64], and weakly electric fish [65], reinforcing the view that robust behavior emerges from dynamic interactions between sensing and movement [54]. Our approach not only advances neuroethological understanding in a non-model system. It also offers conceptual and computational tools for designing biomimetic control strategies in robotics and artificial intelligence.

## Methods

### Animals

*Uloborus diversus* were housed in an on-campus greenhouse at Johns Hopkins University. All animals were transferred to custom indoor habitats and kept on a 12 hr:12 hr light-dark cycle (15–30°C, 50%–70% RH) at least a week before being used for behavioral experiments. Spiders were fed *D. melanogaster* or *D. virilis* once a week. Only adult females were used in this study as adult males do not build orb webs.

### Behavioral assay

Adult females were placed in an arena with a 10 cm x 10 cm perimeter, coated with paper at the edges to encourage web-building. We used a ring of white light LED (Lite-On Inc., LTW-2S3D8) to illuminate spider silk. To increase imaging contrast, a high-absorption background material was placed below the behavioral arena (Acktar, Spectral Black Foil, SB-20x030-1–010). A Photron FASTCAM Mini UX100 high speed camera was set up on the top with a 16 mm fixed-focal length lens (Edmund Optics, #85–865) to record web vibration at 1000 Hz (1280 x 1024 pixel resolution; 0.013 cm/pixel). A side camera (BFS-U3-16S2M-CS USB 3.1 Blackfly, 1440 x 1080 pixel resolution) with a 12 mm fixed-focal length lens (Edmund Optics, #86–570) simultaneously recorded spider’s movements at 100 Hz. PFV (ver.3610) and SpinView (1.27.0.48) software were used to store the recordings in AVI format.

Each web had two recordings: one control and one experimental. In the *Drosophila* vibration experiment, the control condition included an empty web, allowing us to measure baseline vibrations caused by air and terrestrial vibrations. The experimental condition included placing either *D. melanogaster* or *D. virilis* on the web to measure vibrations generated by prey movement on web. In the spider prey capture experiment, the control condition was of a live *U. diversus* on the web without any perturbation. In the experimental condition, either *D. melanogaster* or *D. virilis* was introduced onto the web in the presence of the spider to record its behavioral response.

### Web annotation

We manually annotated 91 spider webs with previously developed an in-house web-tracking Graphical User Interface (GUI) [33]. 80% of the web data was used for training, 15% for validation, and 5% for testing. For each web, we computationally deleted parts of spider web and repeated this process 30 times, helping us to expand our dataset from 91 webs to 2821 webs (= 91 x 31). A U-Net model [38] was trained on this dataset to automatically predict web structure. The model was optimized using a combined binary cross-entropy and Dice loss function, which penalizes both pixel-level prediction errors and poor spatial overlap between the predicted and ground truth masks — a more robust criterion than binary cross-entropy alone for segmenting sparse, thin structures such as spider webs. Model performance was evaluated on a held-out validation set, achieving a Dice coefficient of 0.825, indicating 82.5% overlap between predicted and ground truth web masks. The trained model was subsequently applied to annotate web architecture across all experimental recordings.

### Web vibration analysis

To extract the frequency profile of the web, FFT analysis [30,31,34] was applied on pixel intensities along silk lines. Notably, because our goal was to study web-borne vibrations rather than airborne acoustic signals, we analyzed only time windows after prey were already caught on the web, ensuring that no airborne signals were present during the analyzed periods. The FFT, of a time series with length N, was defined as


$$\begin{array}{c} FFT[k]=\sum_{n=\text{0}}^{N-\text{1}}e^{-\text{2}\pi i\frac{kn}{N}}x[n], \end{array}$$ (1)

where x[n] is the discrete-time signal and k is the frequency bin index. The mean of FFT along all silk lines was computed to investigate whole web vibration.

To compare vibration patterns across all webs, we used fold change defined as


$$\begin{array}{c} \begin{array}{c} Foldchange=\frac{{FFT}_{experimental}}{{FFT}_{control}}. \end{array} \end{array}$$ (2)

We also performed short-time Fourier Transform to investigate the temporal profile of web vibration. It was defined as


$$\begin{array}{c} STFT[m,k]=\sum_{n=\text{0}}^{N-\text{1}}e^{-\text{2}\pi i\frac{kn}{N}}x[n+mR]\cdot \omega [n], \end{array}$$ (3)

where ω[n] is the window function of length 400 samples, m is the frame index, and R = 20 is the hop size (i.e., the step between adjacent frames).

The window length of 400 samples (400 ms at 1000 Hz) was chosen to balance the inherent trade-off between frequency and temporal resolution in the STFT. While a longer window (e.g., 1000 samples) would yield finer frequency resolution (1 Hz), it would reduce temporal resolution to 1000 ms, insufficient to capture the rapid state transitions characteristic of prey capture behavior. A hop size of R = 20 samples was applied between consecutive frames, corresponding to 95% overlap and an effective temporal resolution of 20 ms.

### Validation of vibration imaging by acoustic stimulation

To validate the video-based vibration quantification method, we delivered controlled acoustic stimuli to *U. diversus* webs while recording high-speed video. Pure-tone stimuli (100–500 Hz) were generated using Online Tone Generator (onlinetonegenerator.com) and delivered through a speaker positioned 8 cm from the web. Each frequency was presented at three sound-pressure levels (61.8 ± 0.21, 67.6 ± 0.24, and 71.6 ± 0.35 dB, mean ± s.e.m.; n = 4 webs × 5 frequencies), measured at 8 cm from the speaker using a decibel meter (TopTes TS-501B). Web motion was quantified from pixel-intensity fluctuations along silk threads and analyzed using the fast Fourier transform to obtain amplitude spectra. The 500 Hz stimulus approached the video sampling rate’s Nyquist limit, and consistent with aliasing theory, no resolvable power was expected or observed in the FFT spectra.

### Validation of vibration imaging by piezo stimulation

We used the Physik Instrumente PL128.11 connected with E-650 amplifier to calibrate the displacement sensitivity of our imaging metric, which infers silk motion from optical pixel-intensity fluctuations. As a baseline, we first recorded unstimulated control webs (n = 5). We then drove the piezo at 20 Hz using an Arduino IDE-implemented amplitude sweep triggered through Arduino control scripts executing on a Teensy 3.2.

The PL128.11 piezo has a maximum physical travel of 900 µm, and the drive signal was specified in 8-bit steps (0–255). We delivered seven target amplitude levels (2, 4, 6, 8, 10, 50, and 255 in 8-bit units) at 20 Hz. If the target amplitude level is L, then the input voltage to the amplifier E-650 is


$$\begin{array}{c} \begin{array}{c} V_{in}=\text{1}\text{0}V\times \frac{L}{\text{2}\text{5}\text{5}}. \end{array} \end{array}$$ (4)

The amplifier gain is 6, so amplifier output voltage is


$$\begin{array}{c} \begin{array}{c} V_{out}=\text{6}\times V_{in}=\text{6}\times \text{1}\text{0}V\times \frac{L}{\text{2}\text{5}\text{5}}, \end{array} \end{array}$$ (5)

and converted the digital drive values to nominal physical displacements using the linear mapping:


$$\begin{array}{c} \begin{array}{c} displacement=\frac{\text{9}\text{0}\text{0}\mu m}{\text{6}\text{0}}\times V_{out}=\frac{\text{9}\text{0}\text{0}\mu m}{\text{6}\text{0}}\times \text{6}\times \text{1}\text{0}V\times \frac{L}{\text{2}\text{5}\text{5}}. \end{array} \end{array}$$ (6)

This produced calibrated thread displacements of 7.1, 14.1, 21.2, 28.2, 35.3, 176.5, and 900 µm, confirming that pixel-intensity fluctuations track displacement within the valid detection regime and establish a practical lower-bound sensitivity for sub-pixel silk motion detection.

### Prey regions of interest (ROIs) analysis

To extract prey vibration on the web, we selected regions of interest around *Drosophila*. The prey’s peripheral field radius was twice the radius of the ROI, excluding the central ROI. Both FFT and STFT along silk lines were calculated within ROIs and peripheral field for top camera recordings. For the side camera, FFT and STFT were computed based on the pixel intensities of the fly and silk within the ROIs and the peripheral field, respectively.

The pure STFT prey signal was defined as the STFT within the ROIs, normalized by the peripheral STFT of the prey:


$$\begin{array}{c} \begin{array}{c} pureflysignal=\frac{{STFTpower}_{flycenter(withincyanROI)}}{{STFTpower}_{flyperipheral(outsidecyanROI\&withinmagentaROI)}}. \end{array} \end{array}$$ (7)

To quantify the pure fly signal across 12 top-view recordings, we averaged the STFT power for each behavioral state over time. For side-view recordings, the spatial resolution only permitted detection of vibrations up to 50 Hz. Because *Drosophila* vibrations typically occur below 30 Hz (Fig 1C-1F), we averaged the STFT power within the 0–30 Hz frequency band to examine temporal changes in the pure fly signal. To assess signal dynamics across all recordings, we normalized the duration of each behavioral state prior to averaging across trials. This normalization enabled consistent comparison of pure and raw fly signal evolution during state transitions. Shaded areas in Fig 3E and 3F indicate 95% confidence intervals across recordings.

### *Uloborus diversus’* joint wavelet analysis

We used DeepLabCut [36] to track 20 joints on spider’s anterior and posterior legs: body-coxa, coxa-femur, femur-tibia, and tibia-metatarsus joints, as well as the tip of the tarsus. Since spiders primarily move along the horizontal plane, all horizontal coordinates were centered by subtracting each spider’s centroid. Meanwhile, vertical coordinates were normalized by subtracting their mean value over time.

After centering joint coordinates, we then applied the Morlet continuous wavelet transform [38] to capture spider movements. Wavelet analysis is widely used in behavioral studies [33,38–40] because it captures transient motion patterns that vary over time, enabling the analysis of dynamic and non-stationary animal behaviors.

The wavelet transforms described by Berman et. al [38]. was defined as follows:


$$\begin{array}{c} \begin{array}{c} W_{s,\tau }\left[y(t)\right]=\frac{\text{1}}{\sqrt{s}}\int_{-\infty }^{\infty }y(t)\psi^{*}\left(\frac{t-\tau }{s}\right)dt, \end{array} \end{array}$$ (8)

with


$$\begin{array}{c} \begin{array}{c} \psi (\eta )=\pi^{-\frac{\text{1}}{\text{4}}}e^{i\omega_{\text{0}}\eta }e^{-\frac{\text{1}}{\text{2}\eta^{\text{2}}}}, \end{array} \end{array}$$ (9)

where y(t) is spider’s postural time series, $\omega_{\text{0}}=\text{5}$ is a non-dimensional parameter, τ is a point in time, and s is the time scale of interest as a function of frequency f:


$$\begin{array}{c} \begin{array}{c} s(f)=\frac{\omega_{\text{0}}+\sqrt{\text{2}+{\omega_{\text{0}}}^{\text{2}}}}{\text{4}\pi f}. \end{array} \end{array}$$ (10)

The power spectrum is:


$$\begin{array}{c} \begin{array}{c} S(k,f;\tau )=\frac{\text{1}}{C\left(s(f)\right)}\left|W_{s(f),\tau }\left[y_{k}(t)\right]\right|, \end{array} \end{array}$$ (11)

with the scalar function


$$\begin{array}{c} \begin{array}{c} C(s)=\frac{\pi^{-\frac{\text{1}}{\text{4}}}}{\sqrt{\text{2}s}}e^{\frac{\text{1}}{\text{4}{\left(\omega_{\text{0}}-\sqrt{\text{2}+{\omega_{\text{0}}}^{\text{2}}}\right)}^{\text{2}}}}. \end{array} \end{array}$$ (12)

Finally, the frequency range used was between $f_{min}=\text{0}.\text{1}$ and the Nyquist frequency ($f_{max}=\text{5}\text{0}$ Hz), with 50 frequencies space as follows:


$$\begin{array}{c} \begin{array}{c} f_{i}=f_{max}\cdot {\text{2}}^{-\frac{i-\text{1}}{N_{f}-\text{1}}{\text{log}}_{\text{2}}\frac{f_{max}}{f_{min}}}. \end{array} \end{array}$$ (13)

Wavelet analysis was applied on 20 joints in 4 legs for each side camera recordings. The wavelet spectrum was therefore had a dimension of 20 joints × 50 frequencies × 2 coordinates (vertical and horizontal movements) for each video.

### Stereotyped prey capture behavior motifs

We observed strong correlations among the wavelet representations of all four legs, as well as significant dependence between vertical and horizontal components. Additionally, low-frequency wavelets were primarily associated with noise, such as air current perturbations on the web. To reduce dataset complexity, we focused our analysis on five joint wavelets in the vertical coordinate from the spider’s left anterior leg, using 25 frequencies ranging from 2.38 to 50 Hz. This resulted in a wavelet spectrum of dimensions 5 joints × 25 frequencies × 1 coordinate × 21 recordings.

To identify stereotyped movements during prey-capture, we applied a UMAP [41] for dimensionality reduction to five components, using 100 neighbors. From the wavelet spectrum, we observed 3 different motifs: no movement (static), middle frequency movement (2.38-10 Hz; crouching), and high frequency movement (10–50 Hz). Therefore, K-means clustering [42] with 3 clusters was applied on UMAP to identify 3 different behavioral motifs.

### Unsupervised Hidden Markov Model (HMM)

To characterize spider prey-capture dynamics, we constructed a HMM with three hidden states, initializing the emission probabilities as multivariate Gaussian distributions based on the three motifs identified by K-means clustering. The transition matrix was randomly initialized. The model was then trained using the Baum-Welch algorithm, with a minimum of 50 and a maximum of 500 iterations. Note that true state labels were never used to train HMM. Therefore, the model is unsupervised.

### Generalized linear model (GLM)

We implemented two sensorimotor GLMs to examine reciprocal predator–prey interactions between *U. diversus* and *D. melanogaster*.

For the spider GLM, inputs consisted of two vibratory signals from the fly: (1) pure-fly STFT power, and (2) the fly’s vertical velocity. For the fly GLM, inputs were two vibratory signals from the spider: (1) the mean joint wavelet power across the five anterior-leg joints, and (2) the spider’s vertical velocity. All sensory inputs were Z-scored to normalize across individuals and reduce inter-animal variability. Vertical velocity signals were low-pass filtered using a 25 Hz Butterworth filter to attenuate high-frequency noise.

To estimate linear filters, we adapted the BTA from the spike-triggered average method in the LNP cascade model [50–53]. This approach offers a simple, computationally efficient, and non-parametric means of characterizing stimulus–response mappings for each behavioral state, with the BTA defined as the mean sensory input within a specified 𝜏-length time window preceding the onset of behavioral state 𝑘:


$$\begin{array}{c} {BTA}_{k}=\frac{\text{1}}{N_{k}}\sum_{i=\text{1}}^{N_{k}}s\left(t_{i}-\tau +\text{1}:t_{i}\right), \end{array}$$ (14)

where s(t) is a sensory signal, $t_{i}$ denotes the onset times of state 𝑘, and $N_{k}$ is the number of state transitions.

To extract BTAs, spider behavioral states were defined by a joint-wavelet HMM (Fig 3), whereas fly behavioral states were determined via K-means clustering of pure-fly STFT power. Finally, we projected raw sensory inputs onto the corresponding BTA-derived linear filters (via inner product):


$$\begin{array}{c} \begin{array}{c} x_{purefly}(t)={BTA}_{purefly,k}\cdot s_{purefly}(t), \end{array} \end{array}$$ (15)

$$\begin{array}{c} \begin{array}{c} x_{vy}(t)={BTA}_{vy,k}\cdot s_{vy}(t). \end{array} \end{array}$$ (16)

To estimate the probability of behavioral state 𝑘 given the sensory inputs, a logistic regression model was fit using the BTA-projected signals as predictors (Fig 4B):


$$\begin{array}{c} \begin{array}{c} P\left(z_{t}=k|s(t)\right)=\frac{e^{\left(\beta_{\text{0},k}+\beta_{\text{1},k}x_{purefly}(t)+\beta_{\text{2},k}x_{vy}(t)\right)}}{\sum_{j}e^{\left(\beta_{\text{0},j}+\beta_{\text{1},j}x_{purefly}(t)+\beta_{\text{2},j}x_{vy}(t)\right)}}. \end{array} \end{array}$$ (17)

### Prey localization analysis

We first applied an object detection algorithm to track the spider’s position across all video frames. Radial threads within the spider’s peripheral field were then manually annotated to extract time-varying pixel intensity profiles. The predicted turning angle was defined as the angle between the spider’s body axis (head to abdomen) and the radial thread exhibiting the greatest pixel intensity change in static state prior to movement onset. Of the 12 top-view recordings analyzed, 6 exhibited turning behavior, yielding a total of 10 distinct turning events.

## Supporting information

S1 Fig Validation of video-based spider web vibration measurements using acoustic tones and mechanical perturbation. (A) A 100 Hz sine acoustic tone was delivered to a representative spider web at three calibrated sound pressure levels (61, 67, 71 dB SPL). The acoustic source was positioned 8 cm from the web, with sound intensity verified using a decibel meter placed 8 cm from the speaker. STFT analysis of pixel-intensity fluctuations revealed a robust 100 Hz web-borne vibration, exhibiting monotonic amplitude growth with increasing sound level. (B) Summary of acoustic tone detection (100–500 Hz) across four independent webs. The fold change was quantified as the ratio of FFT amplitude during tone delivery to unstimulated baseline. All webs detected acoustic vibrations, with fold change declining systematically at higher frequencies. (C) Pure tones (100–500 Hz) were applied to a representative web at matched sound levels (61, 67, 71 dB; stimuli marked with a star). The web preserved all driven frequency components, while vibration amplitude in the FFT domain attenuated with rising frequency. The 500 Hz stimulus approached the imaging system’s Nyquist limit (sampling ceiling), and no resolvable spectral power was observed, consistent with sampling theory. (D) Mean Fourier-domain power across four webs tracked input sound amplitude with high fidelity. Error bars denote s.e.m. (E) Piezo-actuated validation of web vibrations using seven programmed drive amplitudes (7.1, 14.1, 21.2, 28.2, 35.3, 176.5, and 900 µm peak-to-peak) at 20 Hz. FFT power from pixel intensity fluctuation increased monotonically with drive amplitude (median Spearman ρ = 1.0; one-sided Wilcoxon test, p = 0.031). Consistent with this trend, FFT power at the control condition was significantly lower than at 7.1 µm (paired one-sided Wilcoxon signed-rank test, p = 0.031), demonstrating that the video-based imaging assay resolves micrometer-scale displacements on the web. The gray trace denotes the mean across five independent webs, and error bars indicate s.e.m. (F) AUC map of the high-frequency band (270–300 Hz) during a *D. melanogaster* trial shows uniformly distributed power. (G) STFT of the same high-frequency (270–300 Hz) range reveals temporally persistent, non-localized noise, suggesting a non-biological origin. (H) Natural frequency experiment using a 3D-printed object on the web (mass = 0.0159 g). Top: mean FFT amplitude in control trials (n = 3). Bottom: mean FFT amplitude across three webs, showing a fundamental frequency at 10 Hz. (I) Fold change heatmap from all three webs. Gentle touches of the object induced the 10 Hz fundamental frequency; stronger perturbations produced harmonics around 20, 30, and 40 Hz.
(EPS)

S2 Fig Comparison of prey-induced inputs across species, and assessment of spider responses. (A) Body mass comparison between *U. diversus*, *D. melanogaster*, and *D. virilis* (n = 24 per group). A one-way ANOVA revealed a significant main effect (F = 29.79, p < 0.0001). Post hoc Mann–Whitney U tests showed significant differences between *U. diversus* (0.0160 ± 0.0025 g, mean ± s.e.m.) and *D. melanogaster* (0.0016 ± 0.0002 g; p < 0.0001), between *D. virilis* (0.0022 ± 0.0002 g) and *D. melanogaster* (p = 0.016), and between *U. diversus* and *D. virilis* (p < 0.0001), but no difference between the two *U. diversus* groups (p = 0.3070). Error bars represent the standard error of the mean (s.e.m.). Left panel: enlarged comparison between *D. virilis* and *D. melanogaster*. (B) Representative FFT spectrum from *D. virilis*, showing broadband low-frequency web vibrations (2–50 Hz), similar to *D. melanogaster*. (C) AUC map along silk threads confirms that *D. virilis*-induced vibrations are spatially localized around the fly’s position. (D) Fold change comparison across 12 webs shows consistent low-frequency vibratory input from *D. virilis* relative to baseline. (E) Representative FFT traces during *U. diversus* interaction with *D. virilis*, illustrating spider-generated harmonic vibrations in rare instances. (F) AUC map from a *D. virilis* trial with spider-induced vibrations, showing widespread vibratory activity across the web. (G) Fold change of spider-generated signals during *D. virilis* trials, showing that harmonics are largely absent in 11 of 12 webs. (H) Spider–prey distance does not differ between *D. melanogaster* and *D. virilis* trials. Each point represents the spider–prey distance at the onset of prey capture for individual trials with *D. melanogaster* (3.28 ± 0.51 cm; mean ± s.e.m., n = 12) and *D. virilis* (2.88 ± 0.25 cm; mean ± s.e.m., n = 12). Horizontal bars indicate mean ± s.e.m. No significant difference was detected between groups (Welch’s t-test, t = 0.694, p = 0.497).
(EPS)

S3 Fig Joint kinematics and HMM-based behavioral state modeling during prey interactions with *D. virilis.* (A) Wavelet spectrograms from joints across all four legs show consistent dynamic signatures during prey capture, justifying the use of the anterior left leg as a representative input for dimensionality reduction and modeling. Ethogram above the spectrogram denotes behavioral states. Abbreviations: Co: Coxa, Tr: Trochanter, Fe: Femur, Pa: Patella, Ti: Tibia, Me: Metatarsus, Ta: Tarsus. (B) Full state transition matrix with *D. melanogaster* interaction from the HMM, including self-transitions, reveals strong within-state persistence and structured inter-state transitions. (C) ΔBIC relative to the previous model shows the greatest improvement between 1 and 3 states, with diminishing gains thereafter. Data are shown as mean  ±  s.d. (filled circles and bars). (D) ΔBIC from the best-fitting model per sequence shows that 1- and 2-state models perform poorly. ΔBIC plateaus beyond 3 states, supporting a three-state model. Dark grey circles represent individual sequences (30 videos). Data are shown as mean  ±  s.d. (filled circles and bars). (E) Joint wavelet data from *D. virilis* interactions projected into the *D. melanogaster*-derived UMAP embedding. (F) Transition matrix from an HMM trained on *D. virilis* data within the shared UMAP space, revealing comparable transition patterns to the *D. melanogaster* model. Left: full transition matrix including self-transitions. Right: normalized matrix with self-transitions excluded. (G) Total counts of each behavioral state did not differ significantly between interactions with *D. melanogaster* and *D. virilis* (Mann–Whitney U test: static, p  =  0.3435; crouching, p  =  0.3639; high-frequency shaking, p  =  0.4837). Data are shown as mean  ±  s.e.m. (bars); colored circles represent the number of behavioral state occurrences in individual recordings. (H) Duration of each behavioral state was significantly shorter during *D. virilis* interactions (Mann–Whitney U test: static, p  =  0.0091; crouching, p  =  0.0121; high-frequency shaking, p  =  0.0015). Data are shown as mean  ±  s.e.m.; colored circles represent individual behavioral events. (I) Dwell time analysis reveals a significantly shorter crouching state during *D. virilis* interactions (p  =  0.0217), while static and high-frequency shaking states showed no significant difference in decay constants between prey types.
(EPS)

S4 Fig Comparison of pure fly vibration signals across behavioral epochs. (A) Example trace showing average STFT power (0–30 Hz) of raw and pure fly signals from representative extended side-view recordings. Pure fly signal power increased during static periods that followed crouching. (B) The pure fly signal in the first static epoch is smaller than in three of the ten subsequent static epochs (p < 0.001), likely because later fly movements involved more rotational than horizontal motion, which our method underestimates. (C) The pure fly signal in the first static epoch is significantly larger than in all crouching epochs (p < 0.001). (D) The pure fly signal in the first static epoch is significantly larger than in all high-frequency shaking epochs (p < 0.001).
(EPS)

S5 Fig Sensory feature selection and temporal window optimization for GLM-based behavioral prediction. (A) Classification performance of spider GLMs using different combinations of sensory features: (1) fly STFT power alone, (2) STFT power with vertical velocity, (3) STFT power with horizontal velocity, and (4) a three-feature model combining STFT power with both vertical and horizontal velocity. Performance was assessed using 25% held-out data and 250 bootstrap iterations (n = 1,000). Filled circles denote mean  ±  s.d. A Kruskal–Wallis test revealed a significant difference among models (p  <  10^-307^). Post hoc Mann–Whitney U tests showed that the two-feature model (STFT + vertical velocity) significantly outperformed the single-feature model (p  <  10^-307^) and the horizontal velocity model (p  <  10^-307^), but did not differ significantly from the three-feature model (p  =  0.683). (B) Classification performance of spider GLMs using different temporal window lengths for filter construction (50 ms, 100 ms, 250 ms, and 500 ms; filled circles denote mean  ±  s.d.). Performance was evaluated as in (A). A Kruskal–Wallis test indicated significant differences across models (p  =  1.989  ×  10^-68^). Post hoc Mann–Whitney U tests revealed that the 100 ms model significantly outperformed both the 50 ms (p  =  1.645  ×  10^-27^) and 500 ms (p  =  1.265  ×  10^-54^) models, and showed a modest but significant improvement over the 250 ms model (p  =  0.0008). (C) Spider BTA filters estimated with alternative window lengths (left: 50 ms; middle: 250 ms; right: 500 ms) show similar structure to the 100 ms filter, demonstrating robustness of the sensory-behavior mapping. (D) Fly BTA filters estimated with alternative window lengths (left: 100 ms; middle: 250 ms; right: 1000 ms) show similar structure to the 250 ms filter.
(EPS)

S1 Video Web vibration signature of *D. melanogaster.* AUC map and corresponding STFT of web-borne vibrations induced by *D. melanogaster* alone.
(MP4)

S2 Video Prey capture behavior: Manual annotation versus HMM classification. UMAP comparison of manually annotated prey capture sequences and behavioral states identified by the HMM framework.
(MP4)

S3 Video Comparison of spider-induced and prey-induced vibratory signals. Spider-modulated STFT signals and pure fly-induced STFT signals on the web.
(MP4)

S4 Video Active modulation of vibratory input by the spider. Demonstration of how spider behavior shapes the STFT profile of prey-induced (raw and pure) vibratory signals during prey capture.
(MP4)

S5 Video Prey localization behavior in *U. diversus.* Spider’s orienting movements in response to prey-induced web vibrations.
(MP4)
