# Supplementary material for: Dynamic vibration-driven feedback shapes predator–prey interactions in an orb-weaving spider
Source: PLoS Comput Biol. 2026 Sep 15;22(9):e1014802. doi: 10.1371/journal.pcbi.1014802 (PMC13592698; doi:10.1371/journal.pcbi.1014802)
Supplement: Supplementary file 1 [file pcbi.1014802.s012.docx]

We are very grateful to the reviewers for their insightful and constructive feedback. Their suggestions have substantially improved the manuscript through the additional clarification throughout the text and revisions to several figures. Our responses to reviewer comments are in red below.

**Reviewer #1: Dynamic vibration-driven feedback shapes predator-prey interactions in orb-weaving spiders**

General Comments:
This is a very sophisticated and thorough documentation, using a highly quantitative approach, of the details of the vibratory dialogue between a predator (an orb weaving spider) and a prey (fruit flies).
- I am not very familiar with this journal and thus cannot discuss its suitability, but this is a very solid study worthy of publication.
- Demonstrating that the spiders are using their web as a 2-way communication platform to manipulate prey behavior to their sensory advantage is not a trivial feat, yet these authors accomplish this with rigorous and convincing data.
- The methods used in this study – e.g., using pixel intensity changes to explore vibration transmission – are very novel and will be of interest to a broad range of scientists.
- The data collection and analyses are very sophisticated and likely to be copied by future researchers, ensuring high citations for this paper.
- Despite the somewhat non-traditional (at least in communication/animal behavior studies) methodological approach, the paper is written in a very understandable fashion, making it highly accessible.
- Overall, the idea that spiders can actively modulate their web to manipulate prey behavior and thus make prey more detectable is a very cool one!

We sincerely thank the reviewer for their thoughtful and encouraging comments. It is gratifying to hear that the methodology and findings resonated, and we genuinely appreciate the time taken to engage so carefully with our work. The suggestions were insightful and pushed us to sharpen both the clarity and presentation of the paper — we believe the manuscript is stronger for it.

Specific Comments

- I would not generalize the title to all orb-weaving spiders but instead say in “AN orb-weaving spider”. While it is likely that this is a general phenomenon of many web-building spiders, it has only been shown in your species so far.

We thank the reviewer for providing this important feedback. We agree that we only tested one spider species in our study and should not over-generalize. We have changed our title from "orb-weaving spiders" to "an orb-weaving spider" in Line 1.

- Abstract, page 2, line 31 – is there a word missing after “increased”?

We thank the reviewer for pointing out the incomplete sentence. We acknowledge that words were missing. We have added "pure fly-induced vibration power" in Line 31.

- Page 15, line 17 – should it be “spiderS exhibit”?

We thank the reviewer for catching this typo. We agree that it should be "spiders exhibit" and have corrected it in Line 329.

- The sample size is not very large for a behavioral study and it might be good to ensure that readers can have confidence in your low sample size (e.g., page 18, line 81; 12 recordings).

We thank the reviewer for raising the potential concern about sample size.

First, we would like to clarify that we have 16 side-view videos in our analysis (Line 389), not 12 as stated in the original manuscript. We have fixed the typo.

Second, although we have 16 recordings, the number of behavioral events analyzed is substantially larger — for example, we identified 22 state transition events from crouching to static (Figure 3E) and 76 events from shaking to crouching (Figure 3F), providing a much larger effective sample size than the number of recordings alone would suggest. We have added text to clarify this point in Line 390 and Line 394. We also added clarification in Figure 3 caption Lines 354-358.

For the top-view videos, we had 12 recordings with totally 6–40 observations per event. We have added the number of events in Lines 371–377. Due to the limited number of recordings, we were unable to observe all behavioral events from the top-view videos. Therefore, we only performed statistical analyses on the side-view videos as discussed above.

- Page 32, line 89 – you should spell out the genus name if it starts a sentence or is a heading

We thank the reviewer for catching this typo. We have corrected it to “*Uloborus diversus*” in Line 714.

**Reviewer #2:**

Hung et al., investigate how U. diversus spiders use web-borne vibrations to guide prey capture behavior. The primary focus in on whether the spider actively modulates its own movements to enhance the detectability of prey-generated signals. The authors use high-speed video recordings to measure vibration, machine-learning-based pose estimation method for tracking the spider’s body posture, and unsupervised behavioral state modeling.
The results show that prey-generated signals from Drosophila are concentrated in the 2-50 Hz range and that species differences arise primarily from vibratory power rather than spectral composition. Moreover, the results discuss three discrete behavioral states and show that these states follow structured transition dynamics during prey capture. Additionally, the results reveal a reciprocal feedback loop between predator and prey. Spider crouching and shaking precede increases in fly-generated vibratory power. Lastly, the results show that spider orientation toward prey is predicted by the radial thread carrying the highest vibratory amplitude. The experiments are logical and the data presented in well-crafted visuals. As such, this work is appropriate for publication. I outline some concerns that are intended to improve the comprehension and follow-up on these impressive efforts.

We sincerely thank the reviewer for this thoughtful and generous summary of our work. It is encouraging to hear that the experimental logic, visualizations, and overall findings were well received.

We genuinely appreciate the time and care taken to engage so thoroughly with the manuscript — the constructive suggestions improved the clarity and presentation of the paper, and we believe the revised manuscript is stronger for it.

Major Points

0. Are three Principal Components really justified based on the eigenvalues? Fig 2A6 and Fig 2D seem quite continuous.

We thank the reviewer for raising this important point. We would like to clarify a terminology issue in the original manuscript: the dimension reduction method used was UMAP (Uniform Manifold Approximation and Projection), not Principal Component Analysis (PCA). Unlike PCA, UMAP is a non-linear method and does not rely on eigenvalue decomposition. We have revised the manuscript to make this distinction clearer (Lines 282-286).

Regarding the apparent continuity in Fig. 2A and Fig. 2D, we agree that the behavioral state space appears continuous rather than discretely clustered, and we believe this accurately reflects the underlying biology. Prey capture behavior in spiders involves smooth transitions between states rather than abrupt switches, and this continuum is a genuine feature of the data rather than an artifact of the analysis. Notably, a similar continuum of behavioral states during prey capture has been reported in zebrafish[1], suggesting this may be a conserved feature across predatory species.

We have added clarifying text to the Results (Lines 282-286) and Discussion sections (Lines 537-540) to explicitly acknowledge the continuous nature of the behavioral spectrum and to explain that our identification of distinct behavioral states represents a practical partition of this continuum for downstream analysis.

1. The authors measure the “pure fly signal” at the fly's location, then use this as the sensory input to the spider GLM. However, the spider is moving (and transitioning between the three states), and its position on the web is not fixed. If the spider systematically moves closer to the fly during or after crouching, then the increased pure fly signal power observed in subsequent static states could reflect reduced propagation distance. Did the authors verify that fly-spider distance did not systematically vary across behavioral states? And if so, that this does not affect the results?

We thank the reviewer for raising this important point. We would like to clarify that the pure fly signal is measured directly at the fly's location, quantifying vibration power caused by fly movements on the web, and is therefore independent of spider position by design.

To further address this concern, we verified that spider-fly distance — normalized to the maximum distance within each video — showed only marginal variation across behavioral states when analyzed per behavioral event, excluding the initial static period prior to the spider's first movement (Kruskal-Wallis H=6.221, p=0.045; static state: mean±std=0.682±0.249, n=22 events; crouching state: mean±std=0.609±0.286, n=111 events; high frequency state: mean±std=0.533±0.275, n=89 events), with large within-state variability reflecting substantial overlap across states.

However, you raise an important point. We primarily used the side-view camera for behavioral analysis because the top-view camera, while capable of acquiring video at a fast frame rate, was only capable of capturing 8 seconds of video. While this allowed us to quantify the vibrational landscape of the web at the beginning of a trial, the majority of the spider’s behavior, including approaching the prey, occurred after 8 seconds of engagement.

Despite this, we were curious about distance effects, and collaborated with a robotics group to explore this in greater detail (Lin et al. [2]). We found that when a spider robot jerks the web, it produces a resonant frequency, but also a second resonant peak along the prey line that increases in frequency as a function of distance. We observed three biological examples of this from our top-view camera, which are included in the Lin et al. manuscript. Whether or not the spider is capable of distinguishing these two frequencies is an open question.

We have added Lines 572-577 in Discussion to clarify.

2. Similarly, does the fly location vary across recordings? Would the fly location have an effect on the results? The web is not a uniform transmission medium (as the paper's own AUC maps show). The fly's position on the web determines which thread segments it directly loads. The spectral content of the signal received at the spider depends not only on the fly's biology (melanogaster vs virilis), but on the mechanical properties of the local thread geometry at the catch site. The authors performed a whole-web FFT averaging, which is a partial mitigation, but the authors should report the catch location, and provide an analysis on whether location variations would affect the results.

We thank the reviewer for pointing out this important point. We fully agree that the vibration signal received by the spider depends not only on fly vibration patterns but also on the mechanical properties of the web.

In Fig. 1H (*D. melanogaster*), the fly location varies across recordings. Despite variation in distance, spiders consistently generate a resonance response in 11 out of 12 recordings when interacting with *D. melanogaster*, but not when interacting with *D. virilis* (1 out of 12 recordings). This consistent species-specific pattern across recordings with different distances suggests that distance alone does not determine the whole web FFT pattern. We added the clarification in Lines 217-220.

Below is an additional figure we could include as a supplemental figure for the paper, however it is important to note that these are different distances across different webs.

We acknowledge that the top-view recordings are limited to 8.73 seconds, which may not capture the full prey-capture dynamics, and that signal propagation may depend on web geometry, which varies across individual webs. Lin et al. [2] used a robophysical model to systematically quantify how spider-prey distance affects the spider's joint vibration patterns at specific prey locations and vibration frequencies. They demonstrated that the resonance profile of the prey-line included the spider-induced resonant frequency, but also a nearby frequency that was a function of prey distance. As mentioned earlier, we had a sparse observation of this due to experimental limitations, which is partly why we turned to using robotics to test hypotheses that were difficult to test in biological systems. We have added this clarification to our manuscript in Lines 572-577.

3. Can the authors provide a hypothesis to why differences in web-borne signals between the two Drosophila species arise primarily from vibratory intensity rather than frequency composition?

We hypothesize that the similar web vibration frequencies induced by the two Drosophila species may stem from comparable grooming frequencies. The flies rarely move their wings, and instead rapidly move their legs in an apparent attempt to remove the silk. Ravbar et al.[3] reported that Drosophila melanogaster grooming occurs at two timescales: 5–7 Hz and 0.3–0.6 Hz. Similarly, Mueller et al. [4] demonstrated that five Drosophila species share core behavioral features, though they differ in the proportions of individual actions. While Drosophila virilis was not included in that study, it likely shares similar grooming behavioral characteristics with Drosophila melanogaster. We have added this in our discussion Line 527-532.

4. The authors normalize the pixel intensity to get “pure” fly (line 323), however, it is unclear how these normalizations were performed. The authors should provide an explicit formulation in the Methods section.

We thank the reviewer for pointing this out. We have added Equation [7] in the Methods section (Line 704) and incorporated it into Line 336.

5. The paper specifies a window of 400 samples for the STFT. Why 400 samples was chosen for the window?

A 400-sample window yields a frequency resolution of 2.5 Hz and a temporal resolution of 400 ms, whereas a 1000-sample window would improve frequency resolution to 1 Hz but at the cost of temporal resolution (1000 ms), which would obscure rapid behavioral transitions.

To enhance temporal precision, we applied a step size of 20 samples (95% overlap) between consecutive windows, yielding an effective temporal resolution of 20 ms — sufficient to resolve the rapid state transitions characteristic of spider prey capture behavior. Although the 95% overlap results in high correlation between adjacent frames, it produces a smooth, continuous temporal representation of the vibration dynamics across the behavioral sequence.

We have added clarification in Line 658-663.

Minor Points

Line 184: The equation is redundant here as it is a repeat of equation 7 in the Methods section. Suggestion: refer on page 9 to equation 7 in the Methods.

We thank the reviewer for pointing this out. We have removed the equation in Line 184 and incorporated it into Method Line 652.

Line 187: Remove: “it is worth noting that”

We thank the reviewer for suggestion. We have removed “it is worth noting that” Line 192.

Line 207-208: It is unclear how Fig 1F shows the statement in this line. Did the authors intend to refer to a different figure.

We thank the reviewer for pointing out this ambiguity. Figure 1F shows representative web vibration recordings under two conditions: the black line represents a control recording from a stationary spider on the web without prey introduction, while the magenta line represents a recording during prey capture behavior following Drosophila introduction. This figure supports the statement in Lines 213-214 by demonstrating the contrast in vibration profiles between baseline and active prey capture conditions. We have revised the figure legend and added clarification in the main text (Line 213-214) to make this connection explicit.

Line 213: The sentence starting with “To confirm that the …” can be a new paragraph.

We thank the reviewer for this suggestion. The sentence beginning with "To confirm that the…" has been moved to a new paragraph. In order to make transition between paragraph smooth, we modified the text in the revised manuscript (Line 221), improving the readability of this section.

Line 226: The concluding findings stated in this paragraph heavily relies on results presented in Fig S2, particularly H-J. For clarity, it might be worth considering moving these figures into the main manuscript as panels for Fig 1.

We thank the reviewer for this suggestion. To improve clarity and readability, we have moved Fig S2H–J to main Figure 1I-K, making this data more immediately visible to the reader. Additionally, we have corrected the colors in Figure S2C and S2F to green to maintain consistency with the species color code used throughout the manuscript.

Line 238: Use “Three” instead of “3”

We thank the reviewer for this suggestion. We have changed "3" to “three” (Line 249).

Line 268: The acronyms throughout the manuscript are not consistent. Multiple acronyms are being defined more than once. Sometimes a term is written as its acronym and others it is fully spelled out. In this line, HMM (268 and 274) is just an example, but this is also present with ROI, FFT, and BTA.

We thank the reviewer for pointing out this inconsistency. We have revised the manuscript to ensure that each acronym is defined only at its first appearance in the main text. For section headings and figure titles, terms are spelled out in full regardless of prior definition, following standard convention. All subsequent appearances use the acronym only.

Corrections have been made at lines 145, 148, 265, 273, 286, 343, 347, 352, 383, 411, 416, 420, 544, 545, 752, 759, 770, 775, 1066, 1091, 1126.

Line 430: Presenting equations 2, 3, 4, and 5 in the main text rather than in the Methods does not add to clarity. Instead, the text feels fragmented. I suggest that the authors move these equations to the Methods section.

We thank the reviewer for this suggestion. Equations 2, 3, 4 and 5 have been moved to the Methods section to improve the flow and readability of the main text. The Results section now refers readers to the Methods for the full mathematical details (Line 780-791).

Line 447: Moreover, equations embedded in text should be treated as text. As such, punctuations should be included. This also applies to the equations in the Methods section.

We thank the reviewer for this correction. We have reviewed all equations throughout the manuscript and added appropriate punctuation. This has been applied consistently across both the main text and the Methods section.

Line 627: Authors use i and j interchangeably to denote sqrt(-1).

We thank the reviewer for this correction. We replaced *j* with *i* to denote sqrt(-1) throughout our manuscript Line 648 and Line 655.

Line 632: Use FFT instead of y. Add information from page 9.

We thank the reviewer point out this important correction. We have change equation 2 to (Line 652):

$$\begin{aligned} Fold change = \frac{{FFT}_{experimental}}{{FFT}_{control}}.\#\left[ 2 \right] \end{aligned}$$

Line 632: The SNR definition presented in equations 1 and 7 is not traditional and differs from the common definition. The authors should provide a citation for this definition or use an alternative name.

We thank the reviewer for this observation. We agree that our definition differs from the conventional SNR. We should use fold change, which is a widely used in biological studies for quantifying the relative difference between experimental and control conditions.

We have renamed this metric as **"fold change"** (Line 652) throughout the manuscript, defined as the ratio of experimental to control vibration amplitude (y_experimental / y_control). We have also modified all SNR to fold change in figures.

Fig 3D: Authors should change the color of one of the green graphs as it is hard to distinguish between the two visually.

We thank the reviewer to point out the visualization issue of Fig 3D. We have changed the darkgreen color to black to improve readability.

Fig 3E: This figure belongs to supplementary more than the main manuscript. Additionally, the authors should include a legend for the three behavioral states

We thank the reviewer to point out this important issue. We have moved Fig 3E to S4A Fig and included legends for three behavioral states.

References:

1.	Mearns DS, Donovan JC, Fernandes AM, Semmelhack JL, Baier H. Deconstructing Hunting Behavior Reveals a Tightly Coupled Stimulus-Response Loop. Current Biology. 2020;30: 54-69.e9. doi:10.1016/j.cub.2019.11.022

2.	Lin EH, Zhou Y, Hung H-Y, Moon L, Gordus A, Li C. Why orb-weaving spiders use leg crouching behavior in vibration sensing of prey on a web: A physical mechanism from robophysical modeling.

3.	Ravbar P, Zhang N, Simpson JH. Behavioral evidence for nested central pattern generator control of Drosophila grooming. doi:10.7554/eLife

4.	Mueller JM, Zhang N, Carlson JM, Simpson JH. Variation and Variability in Drosophila Grooming Behavior. Front Behav Neurosci. 2022;15. doi:10.3389/fnbeh.2021.769372
