# Supplementary material for: Dynamic vibration-driven feedback shapes predator–prey interactions in an orb-weaving spider
Source: PLoS Comput Biol. 2026 Sep 15;22(9):e1014802. doi: 10.1371/journal.pcbi.1014802 (PMC13592698; doi:10.1371/journal.pcbi.1014802)
Supplement: Peer review history

# Peer review: Decision Letter 0

**DOI:** 10.1371/journal.pcbi.1014802.r001

**Authors:** Matthieu Louis, Marieke van Vugt

14 May 2026

PCOMPBIOL-D-26-00652

Dynamic vibration-driven feedback shapes predator–prey interactions in orb-weaving spiders

PLOS Computational Biology

Dear Dr. Gordus,

Thank you for submitting your manuscript to PLOS Computational Biology. After careful consideration, we feel that it has merit but does not fully meet PLOS Computational Biology’s publication criteria as it currently stands. Therefore, we invite you to submit a revised version of the manuscript that addresses the points raised during the review process.

Please submit your revised manuscript by Jul 14 2026 11:59PM. If you will need more time than this to complete your revisions, please reply to this message or contact the journal office at ploscompbiol@plos.org. When you’re ready to submit your revision, log on to https://www.editorialmanager.com/pcompbiol/ and select the 'Submissions Needing Revision' folder to locate your manuscript file.

Please include the following items when submitting your revised manuscript:

* A letter that responds to each point raised by the editor and reviewer(s). You should upload this letter as a separate file labeled 'Response to Reviewers'. This file does not need to include responses to formatting updates and technical items listed in the 'Journal Requirements' section below.

* A marked-up copy of your manuscript that highlights changes made to the original version. You should upload this as a separate file labeled 'Revised Manuscript with Track Changes'.

* An unmarked version of your revised paper without tracked changes. You should upload this as a separate file labeled 'Manuscript'.

If you would like to make changes to your financial disclosure, competing interests statement, or data availability statement, please make these updates within the submission form at the time of resubmission. Guidelines for resubmitting your figure files are available below the reviewer comments at the end of this letter.

As the corresponding author, your ORCID iD is verified in the submission system and will appear in the published article. PLOS supports the use of ORCID, and we encourage all coauthors to register for an ORCID iD and use it as well. Please encourage your coauthors to verify their ORCID iD within the submission system before final acceptance, as unverified ORCID iDs will not appear in the published article. Only the individual author can complete the verification step; PLOS staff cannot verify ORCID iDs on behalf of authors.

We look forward to receiving your revised manuscript.

Kind regards,

Matthieu Louis

Academic Editor

PLOS Computational Biology

Marieke van Vugt

Section Editor

PLOS Computational Biology

**Journal Requirements:**

1) Please ensure that the CRediT author contributions listed for every co-author are completed accurately and in full.

At this stage, the following Authors/Authors require contributions: Hsin-Yi Hung, Abel Corver, and Andrew G Gordus. Please ensure that the full contributions of each author are acknowledged in the "Add/Edit/Remove Authors" section of our submission form.

The list of CRediT author contributions may be found here: https://journals.plos.org/ploscompbiol/s/authorship#loc-author-contributions

2) We ask that a manuscript source file is provided at Revision. Please upload your manuscript file as a .doc, .docx, .rtf or .tex. If you are providing a .tex file, please upload it under the item type u2018LaTeX Source Fileu2019 and leave your .pdf version as the item type u2018Manuscriptu2019.

3) Please upload all main figures as separate Figure files in .tif or .eps format. For more information about how to convert and format your figure files please see our guidelines:

https://journals.plos.org/ploscompbiol/s/figures

4) We notice that your supplementary Figures are included in the manuscript file. Please remove them and upload them with the file type 'Supporting Information'. Please ensure that each Supporting Information file has a legend listed in the manuscript after the references list.

5) Some material included in your submission may be copyrighted. According to PLOS’s copyright policy, authors who use figures or other material (e.g., graphics, clipart, maps) from another author or copyright holder must demonstrate or obtain permission to publish this material under the Creative Commons Attribution 4.0 International (CC BY 4.0) License used by PLOS journals. Please closely review the details of PLOS’s copyright requirements here: PLOS Licenses and Copyright. If you need to request permissions from a copyright holder, you may use PLOS's Copyright Content Permission form.

Please respond directly to this email and provide any known details concerning your material's license terms and permissions required for reuse, even if you have not yet obtained copyright permissions or are unsure of your material's copyright compatibility. Once you have responded and addressed all other outstanding technical requirements, you may resubmit your manuscript within Editorial Manager.

Potential Copyright Issues:

i) Please confirm (a) that you are the photographer of 1B and Supplementary Videoes (S2 to S5), or (b) provide written permission from the photographer to publish the photo(s) under our CC BY 4.0 license.

ii) Figures 1A, and 4A. Please confirm whether you drew the images / clip-art within the figure panels by hand. If you did not draw the images, please provide (a) a link to the source of the images or icons and their license / terms of use; or (b) written permission from the copyright holder to publish the images or icons under our CC BY 4.0 license. Alternatively, you may replace the images with open source alternatives. See these open source resources you may use to replace images / clip-art:

- https://commons.wikimedia.org

- https://openclipart.org/.

Note: If the reviewer comments include a recommendation to cite specific previously published works, please review and evaluate these publications to determine whether they are relevant and should be cited. There is no requirement to cite these works unless the editor has indicated otherwise.

**Reviewers' comments:**

Reviewer's Responses to Questions

Reviewer #1: Dynamic vibration-driven feedback shapes predator-prey interactions in orb-weaving spiders

General Comments:

This is a very sophisticated and thorough documentation, using a highly quantitative approach, of the details of the vibratory dialogue between a predator (an orb weaving spider) and a prey (fruit flies).

- I am not very familiar with this journal and thus cannot discuss its suitability, but this is a very solid study worthy of publication.

- Demonstrating that the spiders are using their web as a 2-way communication platform to manipulate prey behavior to their sensory advantage is not a trivial feat, yet these authors accomplish this with rigorous and convincing data.

- The methods used in this study – e.g., using pixel intensity changes to explore vibration transmission – are very novel and will be of interest to a broad range of scientists.

- The data collection and analyses are very sophisticated and likely to be copied by future researchers, ensuring high citations for this paper.

- Despite the somewhat non-traditional (at least in communication/animal behavior studies) methodological approach, the paper is written in a very understandable fashion, making it highly accessible.

- Overall, the idea that spiders can actively modulate their web to manipulate prey behavior and thus make prey more detectable is a very cool one!

Specific Comments

- I would not generalize the title to all orb-weaving spiders but instead say in “AN orb-weaving spider”. While it is likely that this is a general phenomenon of many web-building spiders, it has only been shown in your species so far.

- Abstract, page 2, line 31 – is there a word missing after “increased”?

- Page 15, line 17 – should it be “spiderS exhibit”?

- The sample size is not very large for a behavioral study and it might be good to ensure that readers can have confidence in your low sample size (e.g., page 18, line 81; 12 recordings).

- Page 32, line 89 – you should spell out the genus name if it starts a sentence or is a heading

Reviewer #2: Hung et al., investigate how U. diversus spiders use web-borne vibrations to guide prey capture behavior. The primary focus in on whether the spider actively modulates its own movements to enhance the detectability of prey-generated signals. The authors use high-speed video recordings to measure vibration, machine-learning-based pose estimation method for tracking the spider’s body posture, and unsupervised behavioral state modeling.

The results show that prey-generated signals from Drosophila are concentrated in the 2-50 Hz range and that species differences arise primarily from vibratory power rather than spectral composition. Moreover, the results discuss three discrete behavioral states and show that these states follow structured transition dynamics during prey capture. Additionally, the results reveal a reciprocal feedback loop between predator and prey. Spider crouching and shaking precede increases in fly-generated vibratory power. Lastly, the results show that spider orientation toward prey is predicted by the radial thread carrying the highest vibratory amplitude. The experiments are logical and the data presented in well-crafted visuals. As such, this work is appropriate for publication. I outline some concerns that are intended to improve the comprehension and follow-up on these impressive efforts.

Major Points

0. Are three Principal Components really justified based on the eigenvalues? Fig 2A6 and Fig 2D seem quite continuous.

1. The authors measure the “pure fly signal” at the fly's location, then use this as the sensory input to the spider GLM. However, the spider is moving (and transitioning between the three states), and its position on the web is not fixed. If the spider systematically moves closer to the fly during or after crouching, then the increased pure fly signal power observed in subsequent static states could reflect reduced propagation distance. Did the authors verify that fly-spider distance did not systematically vary across behavioral states? And if so, that this does not affect the results?

2. Similarly, does the fly location vary across recordings? Would the fly location have an effect on the results? The web is not a uniform transmission medium (as the paper's own AUC maps show). The fly's position on the web determines which thread segments it directly loads. The spectral content of the signal received at the spider depends not only on the fly's biology (melanogaster vs virilis), but on the mechanical properties of the local thread geometry at the catch site. The authors performed a whole-web FFT averaging, which is a partial mitigation, but the authors should report the catch location, and provide an analysis on whether location variations would affect the results.

3. Can the authors provide a hypothesis to why differences in web-borne signals between the two Drosophila species arise primarily from vibratory intensity rather than frequency composition?

4. The authors normalize the pixel intensity to get “pure” fly (line 323), however, it is unclear how these normalizations were performed. The authors should provide an explicit formulation in the Methods section.

5. The paper specifies a window of 400 samples for the STFT. Why 400 samples was chosen for the window?

Minor Points

Line 184: The equation is redundant here as it is a repeat of equation 7 in the Methods section. Suggestion: refer on page 9 to equation 7 in the Methods.

Line 187: Remove: “it is worth noting that”

Line 207-208: It is unclear how Fig 1F shows the statement in this line. Did the authors intend to refer to a different figure.

Line 213: The sentence starting with “To confirm that the …” can be a new paragraph.

Line 226: The concluding findings stated in this paragraph heavily relies on results presented in Fig S2, particularly H-J. For clarity, it might be worth considering moving these figures into the main manuscript as panels for Fig 1.

Line 238: Use “Three” instead of “3”

Line 268: The acronyms throughout the manuscript are not consistent. Multiple acronyms are being defined more than once. Sometimes a term is written as its acronym and others it is fully spelled out. In this line, HMM (268 and 274) is just an example, but this is also present with ROI, FFT, and BTA.

Line 430: Presenting equations 2, 3, 4, and 5 in the main text rather than in the Methods does not add to clarity. Instead, the text feels fragmented. I suggest that the authors move these equations to the Methods section.

Line 447: Moreover, equations embedded in text should be treated as text. As such, punctuations should be included. This also applies to the equations in the Methods section.

Line 627: Authors use i and j interchangeably to denote sqrt(-1).

Line 632: Use FFT instead of y. Add information from page 9.

Line 632: The SNR definition presented in equations 1 and 7 is not traditional and differs from the common definition. The authors should provide a citation for this definition or use an alternative name.

Fig 3D: Authors should change the color of one of the green graphs as it is hard to distinguish between the two visually.

Fig 3E: This figure belongs to supplementary more than the main manuscript. Additionally, the authors should include a legend for the three behavioral states

**********

**Have the authors made all data and (if applicable) computational code underlying the findings in their manuscript fully available?**

The PLOS Data policy requires authors to make all data and code underlying the findings described in their manuscript fully available without restriction, with rare exception (please refer to the Data Availability Statement in the manuscript PDF file). The data and code should be provided as part of the manuscript or its supporting information, or deposited to a public repository. For example, in addition to summary statistics, the data points behind means, medians and variance measures should be available. If there are restrictions on publicly sharing data or code —e.g. participant privacy or use of data from a third party—those must be specified.

Reviewer #1: Yes

Reviewer #2: None

**********

PLOS authors have the option to publish the peer review history of their article (what does this mean?). If published, this will include your full peer review and any attached files.

If you choose “no”, your identity will remain anonymous but your review may still be made public.

**Do you want your identity to be public for this peer review?**  For information about this choice, including consent withdrawal, please see our Privacy Policy.

Reviewer #1: No

Reviewer #2: No

[NOTE: If reviewer comments were submitted as an attachment file, they will be attached to this email and accessible via the submission site. Please log into your account, locate the manuscript record, and check for the action link "View Attachments". If this link does not appear, there are no attachment files.]

**Figure resubmission:**

**Reproducibility:**

?>

# Author comment: Author response to Decision Letter 1

**DOI:** 10.1371/journal.pcbi.1014802.r002

26 Jun 2026

Attachment Submitted filename: Hung_PLOS_Comp_Reviewer_Response_AG.docx

# Peer review: Decision Letter 1

**DOI:** 10.1371/journal.pcbi.1014802.r003

**Authors:** Matthieu Louis, Andrea E. Martin

2 Sep 2026

Dear Dr. Gordus,

We are pleased to inform you that your manuscript 'Dynamic vibration-driven feedback shapes predator–prey interactions in an orb-weaving spider.' has been provisionally accepted for publication in PLOS Computational Biology.

Before your manuscript can be formally accepted you will need to complete some formatting changes, which you will receive in a follow up email. A member of our team will be in touch with a set of requests.

Please note that your manuscript will not be scheduled for publication until you have made the required changes, so a swift response is appreciated.

IMPORTANT: The editorial review process is now complete. PLOS will only permit corrections to spelling, formatting or significant scientific errors from this point onwards. Requests for major changes, or any which affect the scientific understanding of your work, will cause delays to the publication date of your manuscript.

Should you, your institution's press office or the journal office choose to press release your paper, you will automatically be opted out of early publication. We ask that you notify us now if you or your institution is planning to press release the article. All press must be co-ordinated with PLOS.

Thank you again for supporting Open Access publishing; we are looking forward to publishing your work in PLOS Computational Biology.

Best regards,

Matthieu Louis

Academic Editor

PLOS Computational Biology

Marieke van Vugt

Section Editor

PLOS Computational Biology

***********************************************************

Reviewer's Responses to Questions

**Comments to the Authors:**

**Please note here if the review is uploaded as an attachment.**

Reviewer #1: The authors did an excellent job addressing all of the reviewer's concerns.

**********

**Have the authors made all data and (if applicable) computational code underlying the findings in their manuscript fully available?**

The PLOS Data policy requires authors to make all data and code underlying the findings described in their manuscript fully available without restriction, with rare exception (please refer to the Data Availability Statement in the manuscript PDF file). The data and code should be provided as part of the manuscript or its supporting information, or deposited to a public repository. For example, in addition to summary statistics, the data points behind means, medians and variance measures should be available. If there are restrictions on publicly sharing data or code —e.g. participant privacy or use of data from a third party—those must be specified.

Reviewer #1: Yes

**********

PLOS authors have the option to publish the peer review history of their article (what does this mean?). If published, this will include your full peer review and any attached files.

If you choose “no”, your identity will remain anonymous but your review may still be made public.

**Do you want your identity to be public for this peer review?**  For information about this choice, including consent withdrawal, please see our Privacy Policy.

Reviewer #1: No

# Editor report: Acceptance letter

**DOI:** 10.1371/journal.pcbi.1014802.r004

**Authors:** Matthieu Louis, Andrea E. Martin

PCOMPBIOL-D-26-00652R1

Dynamic vibration-driven feedback shapes predator–prey interactions in an orb-weaving spider.

Dear Dr Gordus,

I am pleased to inform you that your manuscript has been formally accepted for publication in PLOS Computational Biology. Your manuscript is now with our production department and you will be notified of the publication date in due course.

The corresponding author will soon be receiving a typeset proof for review, to ensure errors have not been introduced during production. Please review the PDF proof of your manuscript carefully, as this is the last chance to correct any errors. Please note that major changes, or those which affect the scientific understanding of the work, will likely cause delays to the publication date of your manuscript.

Soon after your final files are uploaded, unless you have opted out, the early version of your manuscript will be published online. The date of the early version will be your article's publication date. The final article will be published to the same URL, and all versions of the paper will be accessible to readers.

For Research, Software, and Methods articles, you will receive an invoice from PLOS for your publication fee after your manuscript has reached the completed accept phase. If you receive an email requesting payment before acceptance or for any other service, this may be a phishing scheme. Learn how to identify phishing emails and protect your accounts at https://explore.plos.org/phishing.

Thank you again for supporting PLOS Computational Biology and open-access publishing. We are looking forward to publishing your work!

With kind regards,

Sharmila Kamatchi

PLOS Computational Biology | Carlyle House, Carlyle Road, Cambridge CB4 3DN | United Kingdom ploscompbiol@plos.org | Phone +44 (0) 1223-442824 | ploscompbiol.org | @PLOSCompBiol
